# Cancer‐Like Fragmentomic Characteristics of Somatic Variants in Cell‐Free DNA

**DOI:** 10.1002/advs.202514819

**Published:** 2026-01-22

**Authors:** Zhenyu Zhang, Yunyun An, Mengqi Yang, Yuqi Pan, Xiaoyi Liu, Fanglei Gong, Huizhen Lin, Bianbian Tang, Yunxia Bai, Xin Zhao, Yu Zhao, Changzheng Du, Kun Sun

**Affiliations:** ^1^ Institute of Cancer Research Shenzhen Bay Laboratory Shenzhen China; ^2^ Department of Chemical and Biological Engineering Division of Life Science Hong Kong University of Science and Technology Hong Kong SAR China; ^3^ Shenzhen Medical Academy of Research and Translation Shenzhen China; ^4^ School of Life Sciences Westlake University Hangzhou China; ^5^ Hepato‐Biliary Surgery Division Shenzhen Third People's Hospital The Second Affiliated Hospital Southern University of Science and Technology Shenzhen China; ^6^ Molecular Cancer Research Center School of Medicine Shenzhen Campus of Sun Yat‐sen University Sun Yat‐sen University Shenzhen China; ^7^ Cancer Center Beijing Tsinghua Changgung Hospital School of Clinical Medicine Tsinghua Medicine Tsinghua University Beijing China

**Keywords:** artificial intelligence, cancer diagnosis, end motif, epigenomics, liquid biopsy

## Abstract

Cell‐free DNA (cfDNA) in plasma consists of short DNA fragments resulting from a non‐random fragmentation process, with distinct fragmentomic characteristics that are related with their cellular origins. Here, we report that somatic variant signatures in cfDNA markedly differ between non‐cancerous controls and cancer patients, indicating that tumor‐associated signals are retained in these variants. Surprisingly, even in controls, cfDNA molecules harboring somatic variants exhibit cancer‐like fragmentomic characteristics, such as reduced size, decreased DNA methylation, and altered end motif usages and distributions in the nucleosome structure. Further investigations suggest that such cancer‐like traits are associated with somatic variants derived from clonal hematopoiesis. Importantly, these somatic variants‐associated fragmentomic aberrations are more pronounced in cancer patients, enabling cancer diagnosis. In a large pan‐cancer cohort, we utilize AI to integrate genomic, fragmentomic, and epigenomic features to develop diagnostic models named FreeSV and FreeSV+. Leveraging somatic variant‐associated features alone, the FreeSV model achieved area under the ROC curves (AUCs) between 0.81–0.92 across cancer types; however, when genomewide features are also included, the AUCs of FreeSV+ model substantially increased to 0.93–0.99 across cancer types, highlighting the significance of integrative genomic and fragmentomic analyses in cfDNA for cancer liquid biopsy.

## Graphical abstract

We report that in non‐cancerous subjects, cell‐free (cfDNA) molecules harboring somatic variants exhibit cancer‐like fragmentomic characteristics associated with clonal hematopoiesis. Importantly, these somatic variant‐associated fragmentomic aberrations are more pronounced in cancer patients. Leveraging such somatic variant‐associated signals in cfDNA, we develop FreeSV and FreeSV+ models and validate them in a large‐scale cohort, which demonstrates high performance for pan‐cancer diagnosis.

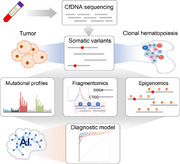

## 1 Introduction

Circulating cell‐free DNA (cfDNA) molecules in human plasma are small fragments of DNA that originate from dead cells (e.g., through apoptosis or necrosis) [1]. In non‐cancerous subjects, cfDNA is primarily sourced from the hematopoietic system and liver [2, 3, 4]; in contrast, in cancer patients, the tumors release cfDNA into plasma pool [5], which serves as a valuable analyte for non‐invasive diagnostics, a process commonly referred to as “liquid biopsy” [6]. Tumor‐derived cfDNA not only carry somatic mutations but also retain epigenomic modifications such as DNA methylation [7, 8]. Furthermore, recent studies indicate that the fragmentation patterns, termed “fragmentomics”, of tumor‐derived cfDNA differ from those of non‐tumor‐derived cfDNA. These patterns are influenced by endonuclease activities and preferences, including shorter fragment sizes [9], altered end or breakpoint motif usages [10, 11], and increased fractions of ends cut within nucleosomes [12, 13], all of which have gained substantial attention as emerging biomarkers in cancer liquid biopsy [8, 14, 15, 16].

In most cancer patients, tumor‐derived cfDNA usually account for a small proportion of the total cfDNA pool in plasma, posing significant challenges for accurately identifying tumor‐derived somatic mutations. To overcome this limitation, one widely utilized strategy is to sequence cfDNA at extra‐high depth, either through whole genome sequencing or targeted capture [17, 18, 19]. For instance, in our previous study involving a hepatocellular carcinoma (HCC) patient, we sequenced cfDNA to approximately 220‐fold human haploid genome coverage, alongside genotyping paired white blood cells to profile and filter out germline variants [19]. Recent advancements in computational algorithms, such as cfSNV, necessitate at least 200‐fold coverage to detect mutations present at a 5% frequency in cfDNA [20]. In contrast, most current liquid biopsy studies, particularly those focusing on cfDNA fragmentomics [8, 14], typically generate data with less than five‐fold human haploid genome coverage, often lacking paired germline genotypes. Additionally, various factors like germline polymorphisms, technical artifacts (including PCR and sequencing errors), aging, and clonal hematopoiesis introduce variants into cfDNA [21, 22, 23, 24]. It is estimated that up to ∼18% of blood cells may harbor clonal hematopoiesis‐associated mutations [25]. In non‐cancerous individuals, clonal hematopoiesis constitutes a major source of somatic variants in cfDNA, and clonal hematopoiesis‐derived somatic mutations are readily detectable in cfDNA [22, 23, 24, 26]. Yet, the characteristics of clonal hematopoiesis‐derived somatic variants in cfDNA have not been fully profiled.

Interestingly, large‐scale cancer studies, such as The Cancer Genome Atlas (TCGA), have revealed that somatic mutations in tumors exhibit non‐random patterns, known as “mutational signatures”. The COSMIC (Catalogue of Somatic Mutations in Cancer) database has catalogued a curated list of mutational signatures in pan‐cancer, along with annotations pertaining to cancer types and associated pathologies [27, 28]. A recent investigation by Wan et al. demonstrated that the mutational signatures in cfDNA from cancer patients differ from those of controls, thereby showing potential in cancer diagnosis [29]. Additionally, Bruhm et al. reported genome‐wide alterations in mutation frequencies and types among cancer patients [30], underscoring the merit of analyzing somatic variants within low‐pass cfDNA datasets. Given the substantial volume of low‐pass cfDNA data generated in cancer liquid biopsy studies, including those generated via whole genome bisulfite sequencing [31], Enzymatic Methyl‐seq (EM‐seq) [32] or similar protocols, analyzing somatic variants might yield novel biomarkers that enhance diagnostic performance and provide insights into cfDNA biology, particularly regarding fragmentomic characteristics linked to somatic variants that remain unexplored. In this study, we examine the characteristics of somatic variants in low‐pass cfDNA without paired germline genotypes. Beyond mutational signatures, we further investigate the cfDNA fragmentomics and epigenomics associated with variants and assess their potential in constructing cancer diagnostic models.

## 2 Results

### 2.1 Schematic Workflow for Genomic and Fragmentomic Characterizations of Somatic Variants

**FIGURE 1:**
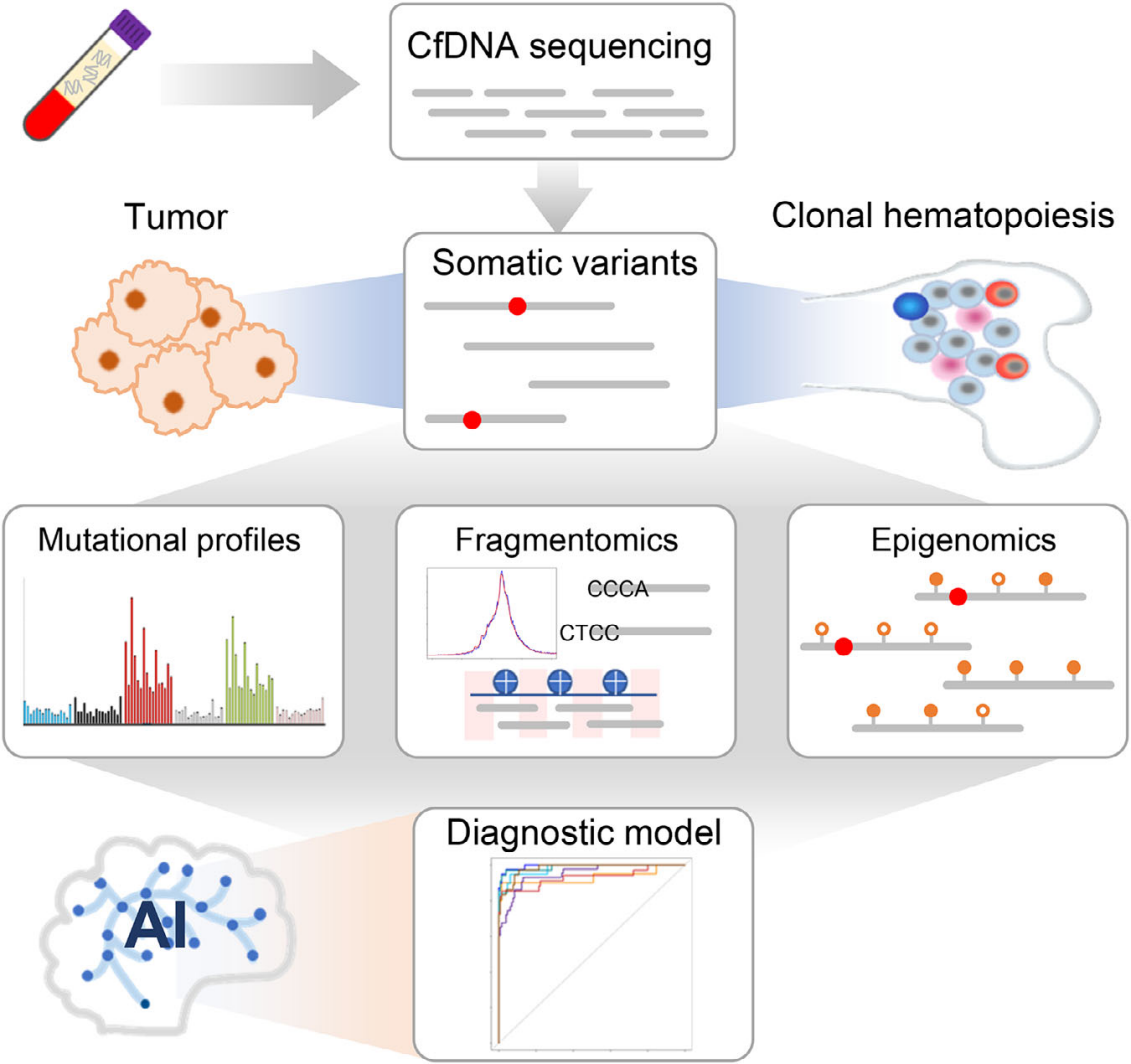
Schematic workflow of somatic variant analysis in cfDNA. We extracted somatic variants from low‐pass cfDNA data without paired germline genotypes, which might originate from clonal hematopoiesis and tumors. We then investigated the genomic, fragmentomic, and epigenomic features associated with the somatic variants to identify tumor‐associated signals. Lastly, we incorporated artificial intelligence these features to build cancer diagnostic models.

Figure 1 showed the schematic workflow of this study. For each low‐pass cfDNA whole genome sequencing data, we identified somatic variants in the absence of germline genotyping information. A stringent filtering strategy was employed, utilizing data quality metrics and known polymorphism sites (see Methods). We then profiled and compared the mutational signatures between controls and cancer samples to elucidate tumor‐associated footprints within the somatic variants, focusing specifically on single‐base substitutions (SBSs). Subsequently, we extracted cfDNA molecules encompassing the somatic variants and examined their fragmentomic and epigenomic characteristics (for EM‐seq data only) and compared them to those covering reference alleles of the same loci. Lastly, we leveraged artificial intelligence (AI) to integrate the genomic, fragmentomic, and epigenomic features of cfDNA somatic variants, facilitating the development of cancer diagnostic models aimed at exploring the translational value of somatic variants in cfDNA.

### 2.2 Genomic and Fragmentomic Profiles of Somatic Variants in Low‐Pass cfDNA Data

**FIGURE 2:**
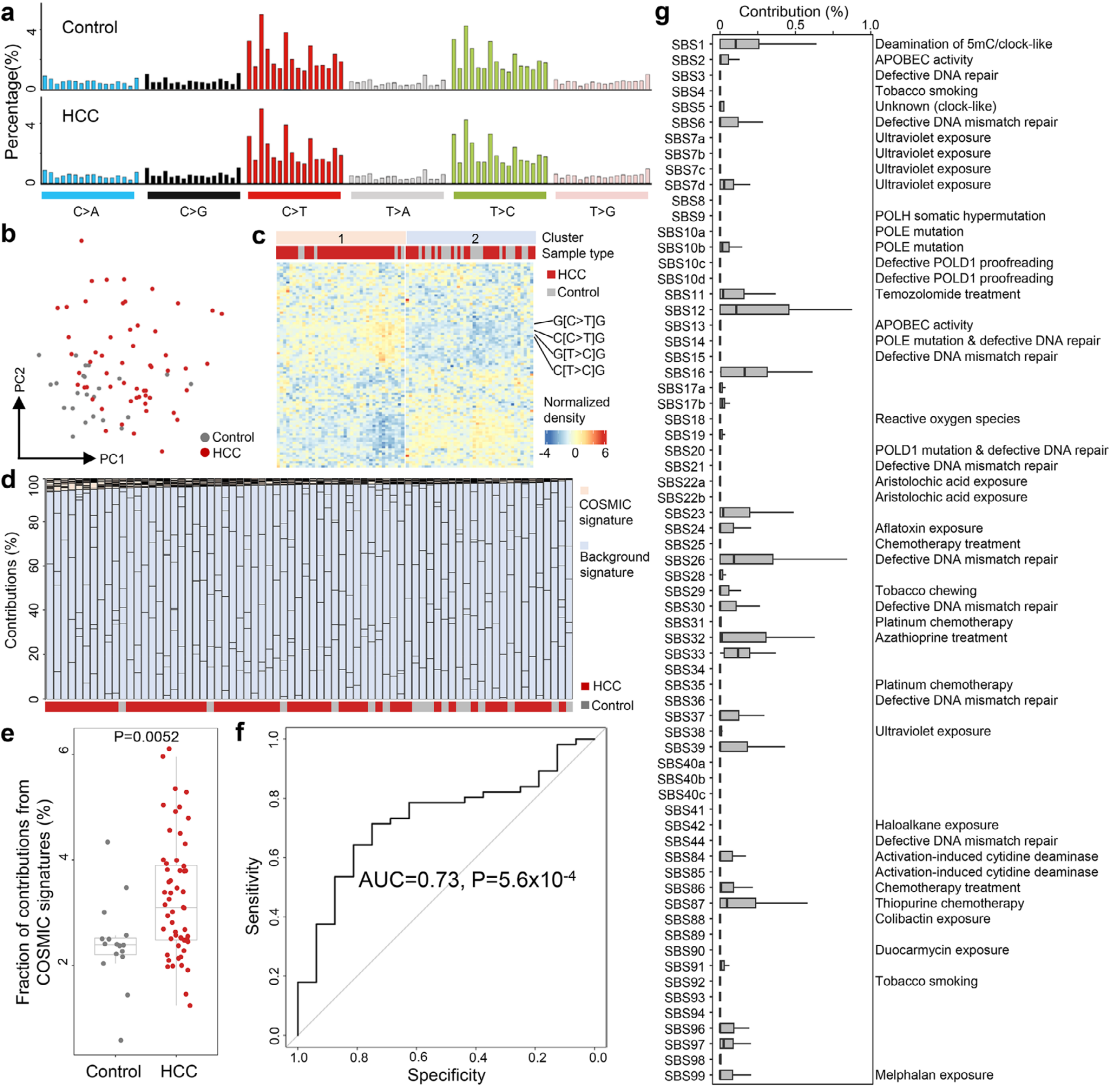
Genomic profiles of somatic variants in cfDNA in our Hepatocellular Carcinoma (HCC) cohort. (a) Mutation profiles of a non‐cancerous control and an HCC sample. (b) Principal Component Analysis and (c) unsupervised clustering results based on mutation profiles. (d) Deconvolution results against a pool of COSMIC mutational signatures and “background signatures” from controls. (e) Total contributions from COSMIC mutational signatures between controls and HCC samples after deconvolution. *P*‐value was calculated using Mann–Whitney U test. (f) Receiver Operating Characteristic curve analysis for differentiating HCC patients from controls using contributions from COSMIC mutation signatures after deconvolution. *P*‐value was calculated using *Z*‐test. (g) Contributions of COSMIC mutational signatures in HCC samples. In b,e, each dot represents one sample. In e,g, boxplots represent the median, upper and lower quartiles and whiskers indicate 1.5 × IQR.

We first examined a cohort of hepatocellular carcinoma (HCC) samples from our previous study, which included 56 HCC samples and 24 age‐ and gender‐matched non‐cancerous controls [12]. All cfDNA samples in this cohort were sequenced to ∼3‐5‐fold human haploid genome coverage (Figure S1a). Following stringent filtering, we obtained 100 000–150 000 somatic variants (i.e., candidate mutations) for each sample. Notably, while there were no statistical differences in sequencing depths between HCC and control samples, the number of somatic variants were significantly higher in HCC samples compared to controls at equivalent sequencing depths (P = 1.7 × 10^−4^, Chow test; Figure S1b,c). We then calculated frequencies of the mutation types according to sequence context for each sample. The results indicated that mutation profiles were generally similar between HCC and control samples, with increased frequencies in C>T and T>C transitions (Figure 2a and. Table S1). However, Principal Component Analysis (PCA) and unsupervised clustering both revealed systematic differences between HCC samples and controls (Figure 2b,c). As depicted in Figure 2c, the samples clustered into two groups: 35 out of 40 samples were HCC in Cluster‐1, which was significantly higher than Cluster‐2 where only 21 out of 40 samples were HCC (P = 0.0015, Chi‐Squared test). Furthermore, the top‐ranked mutation contexts significantly elevated in the HCC‐enriched Cluster‐1, including C[C>T]G, G[C>T]G, C[T>C]G, and G[T>C]G (all *P* < 10^−5^, Mann–Whitney U tests with Benjamini & Hochberg adjustment), have been previously reported as common mutation types in HCC and various cancers [28, 33].

To further investigate the biological implications of the mutation profiles, we deconvoluted them against known tumor mutational signatures. Given that tumor‐derived DNA constituted only a small fraction of the cfDNA pool, besides the 67 mutational signatures for single‐base substitutions documented in the COSMIC database (excluding signatures annotated as “sequencing artifacts”), we also randomly selected eight control samples to model the mutation patterns typical of non‐cancerous backgrounds, which may arise from technical artifacts and clonal hematopoiesis. For all samples, high cosine similarities (>0.999 in all samples; Figure S2a) were achieved between the original mutation profiles and the fitting results, confirming successful deconvolutions [29]. As expected, both HCC and control samples predominantly reflected contributions from the “background signatures” (Figure 2d). However, HCC samples exhibited significantly higher contributions from COSMIC mutational signatures compared to controls (P = 0.0052, Mann–Whitney U test; Figure 2e). Receiver operating characteristic (ROC) curve analysis indicated that the total contributions of COSMIC mutational signatures effectively differentiated HCC samples from controls, with an area under the ROC curve (AUC) value of 0.73 (P = 5.6 × 10^−4^, Z‐test; Figure 2f). Additionally, the total contributions of COSMIC mutational signatures displayed a positive correlation with the tumor DNA fractions in HCC samples (P = 6.8 × 10^−4^, Spearman correlation; Figure S2b). Of the 67 COSMIC mutational signatures, SBS1, SBS12, SBS16, SBS26, and SBS33 showed notable contributions in HCC samples (Figure 2g), with SBS1, SBS12, and SBS16 previously recognized as commonly present in HCC [28]. Collectively, these findings suggest that the mutation profiles in shallow depth cfDNA samples contain substantive tumor‐associated information beyond mere background noises, warranting further investigations.

### 2.3 Fragmentomic Features of cfDNA Harboring Somatic Variants

**FIGURE 3:**
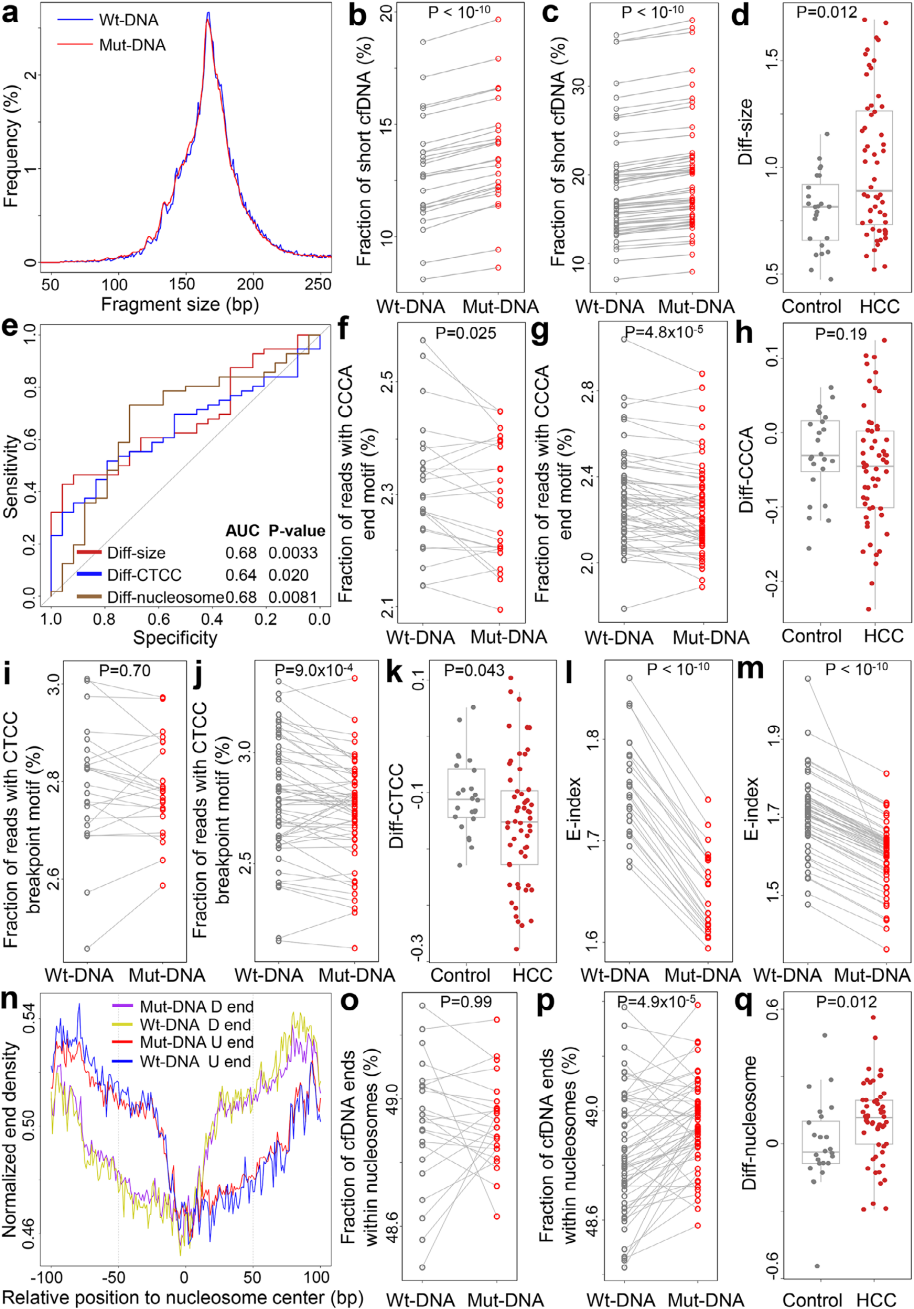
Fragmentomic features of cfDNA associated with somatic variants in the HCC cohort. (a) Size distributions of cfDNA compassing variant (Mut‐DNA) and reference (Wt‐DNA) alleles in the variant loci in an HCC sample. (b,c) Fraction of short cfDNA fragments (defined as shorter or equal to 150 bp) between Wt‐ and Mut‐DNA in b) control subjects and c) HCC patients. (d) Differences in fractions of short fragments between Mut‐ and Wt‐DNA (Diff‐size) between controls and HCC samples. (e) ROC curves for differentiating HCC patients from controls using various fragmentomic features. (f,g) Fraction of reads with 5’‐CCCA end motif for Wt‐ and Mut‐DNA in (f) controls and (g) HCC samples. (h) Differences in fraction of reads with 5’‐CCCA end motif (Diff‐CCCA) between Mut‐ and Wt‐DNA in controls and HCC samples. (i,j) Fraction of reads with CT‐5’‐CC breakpoint motif for Wt‐ and Mut‐DNA in (i) controls and (j) HCC samples. (k) Differences in fraction of reads with CT‐5’‐CC breakpoint motif (Diff‐CTCC) between Mut‐ and Wt‐DNA in controls and HCC samples. (l,m) E‐index values for Wt‐ and Mut‐DNA in (l) controls and m) HCC samples. (n) Orientation‐aware fragment end distributions in the nucleosomal context in an HCC sample. U and D end stand for Upstream and Downstream end, respectively. (o,p) Fraction of ends located within nucleosomes (defined as ± 50 bp from the nucleosome center) for Wt‐ and Mut‐DNA in (o) controls and (p) HCC samples. (q) Differences in fraction of ends located within nucleosomes (Diff‐nucleosome) between Mut‐ and Wt‐DNA in controls and HCC samples. In (b,c,f,g,i,j,l,m,o,p), *P*‐values were calculated using paired *t*‐tests. In (d,h,k,q), P‐values were calculated using Mann–Whitney U test. In (e), P‐values were calculated using *Z*‐tests. In (b–d,f–m,o–q), each dot represents one sample. In (d,h,k,q), boxplots represent the median, upper and lower quartiles and whiskers indicate 1.5 × IQR.

Next, we examined the fragmentomic characteristics associated with the somatic variants identified in our HCC cohort. For all loci with somatic variants, we extracted cfDNA reads harboring the variant alleles (referred to as “Mut‐DNA” hereafter) alongside those covering reference alleles (referred to as “Wt‐DNA”). We observed that, for both controls and HCC samples, Mut‐DNA fragments were shorter than Wt‐DNA, as indicated by significantly higher fractions of short fragments (i.e., ≤150 bp; both *P* < 10^−10^, paired *t*‐tests; Figure 3a‐c). We quantified the difference in fractions of short fragments between Mut‐ and Wt‐DNA for each sample, termed “Diff‐size”. As shown in Figure 3d, Diff‐size values were significantly greater in HCC samples than in controls (P = 0.012, Mann–Whitney U test), with an AUC value of 0.68 for differentiating the two groups (P = 0.0033, Z‐test; Figure 3e). We then investigated two well‐studied motif features: the 5’‐CCCA end motif and the CT‐5’‐CC breakpoint motif (Figure 3f–k) [10, 11, 34]. The 5’‐CCCA end motif exhibited a significant reduction in Mut‐DNA among controls, while no significant differences were found for the CT‐5’‐CC breakpoint motif (P = 0.025 and 0.70, respectively, paired *t*‐tests). As contrast, both motifs were significantly underrepresented in Mut‐DNA from HCC samples (both *P* < 0.001; paired *t*‐tests). To quantify the differences in motif usage between Mut‐ and Wt‐DNA, we defined “Diff‐CCCA” and “Diff‐CTCC”. We found a significant difference in Diff‐CTCC between HCC and controls, while it was not statistically different for Diff‐CCCA (P = 0.043 and 0.19, respectively, Mann–Whitney U tests). Moreover, Diff‐CTCC exhibited an AUC value of 0.64 for distinguishing HCC samples from controls (P = 0.020, *Z*‐test; Figure 3e).

Lastly, we investigated the distribution of fragment ends in both Mut‐ and Wt‐DNA. Notably, the E‐index values, a metric assessing the consistency of fragment ends in a given sample compared to a panel of healthy controls [1], were significantly lower for Mut‐DNA than for Wt‐DNA in both control and HCC samples (both *P* < 10^−10^, paired *t*‐tests; Figure 3l,m). This finding indicates a greater discordance between the ends of Mut‐DNA and those from healthy subjects. However, there was no statistically significant difference in the extent of E‐index decrease between these two groups (Figure S3). We further profiled the end distributions of Mut‐ and Wt‐DNA within the nucleosomal context of GM12878 cells (lymphoid lineage), a commonly used nucleosome track in cfDNA fragmentomic analyses, in an orientation‐aware manner [1, 12, 35, 36, 37]. As shown in Figure 3n–p, Mut‐DNA exhibited a higher fraction of ends located within nucleosomes (defined as ±50 bp from the nucleosome center) compared to Wt‐DNA in HCC samples (P = 4.9 × 10^−5^, paired *t*‐tests), but such difference was not observed in controls. Additionally, we quantified the difference in the fraction of ends located within the nucleosomes between Mut‐ and Wt‐DNA, referred to as “Diff‐nucleosome”. This analysis revealed significant differences between HCC samples and controls (P = 0.012, Mann–Whitney U test; Figure 3q), and Diff‐nucleosome demonstrated an AUC value of 0.68 for differentiating the two groups (P = 0.0081, *Z*‐test; Figure 3e). Collectively, these results suggest that cfDNA encompassing somatic variants exhibit cancer‐like fragmentomic features, even in non‐cancerous individuals. Furthermore, the pronounced differences in fragmentomic characteristics were more evident in HCC samples, indicating their potential as biomarkers for cancer diagnosis.

### 2.4 Somatic Variant‐Associated cfDNA Fragmentomics in Mouse Model

**FIGURE 4:**
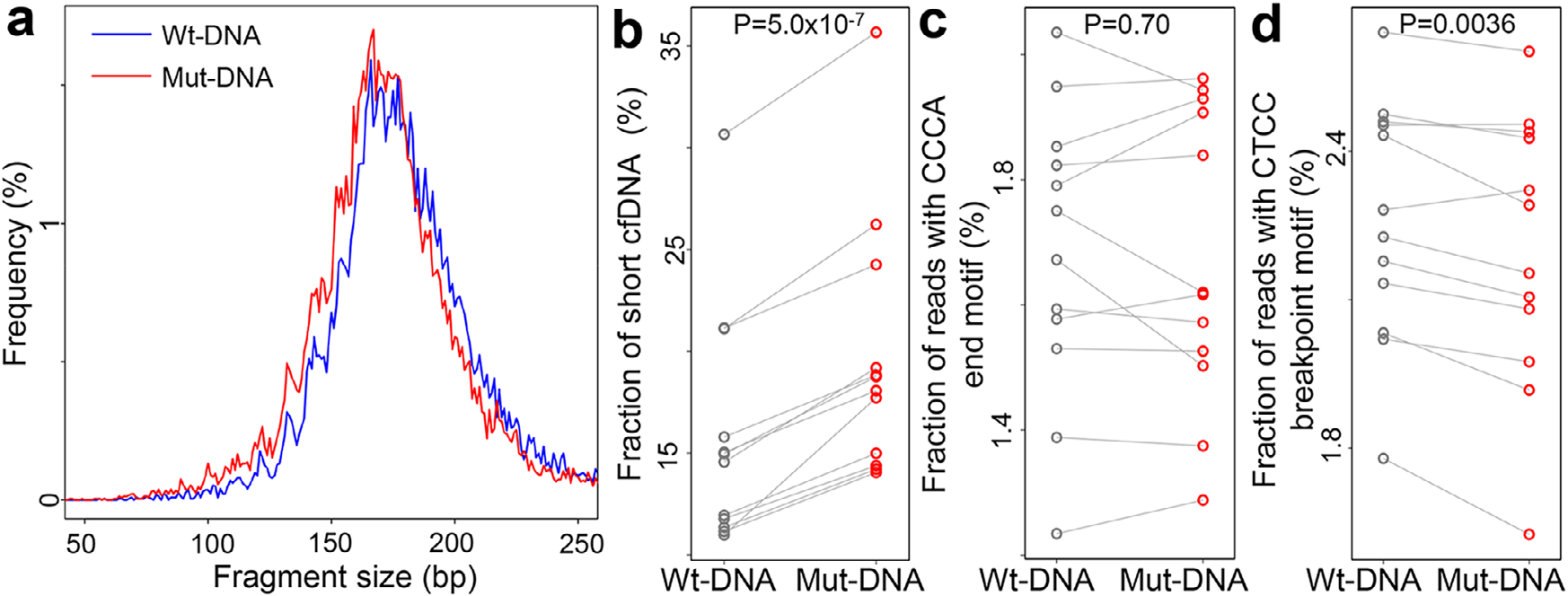
Somatic variant‐associated cfDNA fragmentomics in murine samples (N = 12). (a) Size distributions of cfDNA compassing variant (Mut‐DNA) and reference (Wt‐DNA) alleles in one sample. Comparisons in fractions of (b) short cfDNA fragments, (c) reads with 5’‐CCCA end motif, and (d) reads with CT‐5’‐CC breakpoint motif between Wt‐DNA and Mut‐DNA. In (b–d), *P*‐values were calculated using paired *t*‐tests.

To validate the cancer‐like fragmentomic features of somatic variants in control subjects, we collected and analyzed cfDNA samples from C57BL/6J strain mice (N = 12). As these mice are genetically identical to the reference genome of this strain [38], all detected somatic variants in cfDNA were attributed to clonal hematopoiesis or technical artifacts rather than germline polymorphisms. We identified somatic variants in these samples and extracted cfDNA molecules covering somatic variants (i.e., Mut‐DNA) as well as those covering reference alleles at the same loci (i.e., Wt‐DNA), following the approach used in human data analysis. The size distribution of Mut‐ and Wt‐DNA from a representative murine cfDNA sample was illustrated in Figure 4a. Consistent with the human data, Mut‐DNA showed significantly shorter size compared to Wt‐DNA (P = 5.0 × 10^−7^, paired t‐test; Figure 4b). Moreover, we did not observe significant differences in 5’‐CCCA end motif usage between Mut‐ and Wt‐DNA (P = 0.70, paired *t*‐test; Figure 4c); however, Mut‐DNA exhibited a significant reduction in CT‐5’‐CC breakpoint motif usage compared to Wt‐DNA (P = 0.0036, paired t‐test; Figure 4d). Collectively, these findings conformed alterations in cfDNA fragmentomics associated with Mut‐DNA in non‐cancerous controls.

### 2.5 Fragmentomic Characteristics of Clonal Hematopoiesis‐Derived cfDNA

**FIGURE 5:**
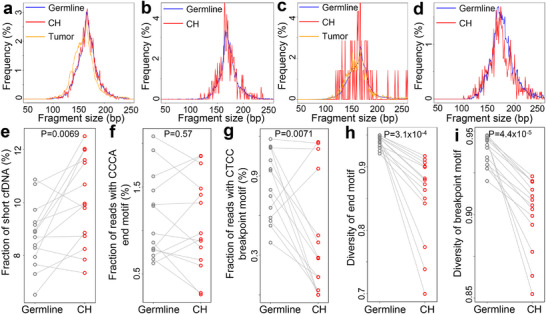
Fragmentomic characteristics of clonal hematopoiesis‐derived cfDNA. (a–c) Size distributions of cfDNA fragments covering tumor‐ and clonal hematopoiesis‐derived somatic variants as well as the germline alleles in (a) liver cancer, (b) colorectal cancer, and (c) breast cancer patients. Note that tumor‐derived somatic variants were not detected in cfDNA of the colorectal cancer patient. (d–i) in a non‐cancerous cohort, (d) size distributions of cfDNA fragments covering clonal hematopoiesis‐derived somatic variants or the corresponding germline alleles in one representative sample; and comparisons in fractions of (e) short cfDNA fragments, (f) reads with CCCA end motif, (g) reads with CTCC breakpoint motif, (h) diversity of end motifs, and (i) diversity of breakpoint motifs between cfDNA molecules covering clonal hematopoiesis‐derived somatic variants and the corresponding germline alleles. CH: clonal hematopoisis. In (e–i), *P*‐values were calculated using paired *t*‐tests.

Given that technical errors in sequencing data are stochastic [39], the cancer‐like fragmentomic characteristics might be associated with clonal hematopoiesis. To explore this hypothesis, one hepatocellular carcinoma was recruited, and we collected the pre‐operative blood sample and tumor tissue. Peripheral blood mononuclear cells (PBMCs) and plasma cfDNA were subsequently isolated from blood. By genotyping both the PBMCs and tumor, we identified somatic variants that were uniquely present either in PBMCs (i.e., clonal hematopoiesis‐derived) or tumor (i.e., tumor‐derived). These variant loci were then interrogated in the cfDNA. As a result, we identified 3,778 and 93,923 cfDNA fragments covering clonal hematopoiesis‐ and tumor‐derived variants, respectively. The proportion of short fragments among molecules carrying clonal hematopoiesis‐ and tumor‐derived variants were 19.2% and 31.6%, respectively, both of which were significantly higher than the proportion in cfDNA fragments covering germline alleles (17.9%; Figure 5a). To validate the result, we recruited another patient suffering from colorectal cancer and collected the tumor tissue and post‐operative blood samples. Notably, cfDNA reads covering tumor‐specific variants were virtually absent, indicating minimal to no contribution of tumor‐derived cfDNA in this sample. In contrast, we successfully identified 340 cfDNA reads harboring clonal hematopoiesis‐derived somatic variants. As shown in Figure 5b, the proportion of short fragments among molecules carrying clonal hematopoiesis‐derived variants was 11.8%, much higher than the corresponding proportion in cfDNA fragments covering germline alleles (8.8%). Consistent findings were observed in an external breast cancer sample from Butler et al. study [40] with both PBMC and tumor genotypes available. In this case, the proportions of short cfDNA fragments were 28.6% for clonal hematopoiesis‐derived variants, 31.6% for tumor‐derived variants, and 26.3% for germline alleles, respectively (Figure 5c), further supporting an overall tendency toward shorter fragment lengths in cfDNA molecules carrying clonal hematopoiesis‐derived somatic variants.

To validate these observations, we further analyzed a public dataset from Nix et al. [41], which included seven non‐cancerous individuals who underwent targeted capture sequencing of cfDNA (two biological replicates per subject; total N = 14 samples) and matched PBMCs. In the high‐depth PBMC data (>1000‐fold coverage), germline single‐nucleotide polymorphisms (SNPs) typically exhibit allele frequencies near 50%. Therefore, variants with minor allele frequencies significantly below 50% were classified as clonal hematopoiesis‐derived, while the corresponding major alleles were considered germline [41]. Consistent with the cancer patient samples, cfDNA fragments carrying clonal hematopoiesis‐derived somatic variants were significantly shorter than those carrying germline alleles (P = 0.0069, paired t‐test; Figure 5d,e). Additionally, the sufficient cfDNA reads with clonal hematopoiesis‐derived variants in this dataset enabled further examination of their motif features. As shown in Figure 5f–i, cfDNA molecules harboring clonal hematopoiesis‐specific variants exhibited aberrant end‐motif profiles, including reduced usage of the CT‐5’‐CC breakpoint motif and decreased motif diversities. Given that tumor‐derived cfDNA is characterized by both shorter fragment lengths and altered end‐motif patterns [9, 10, 11], our findings suggest that clonal hematopoiesis may similarly influence cfDNA fragmentation, producing cancer‐like fragmentomic signatures.

### 2.6 Fragmentomic Features of Somatic Variants in cfDNA in Other Cancer Types

**FIGURE 6:**
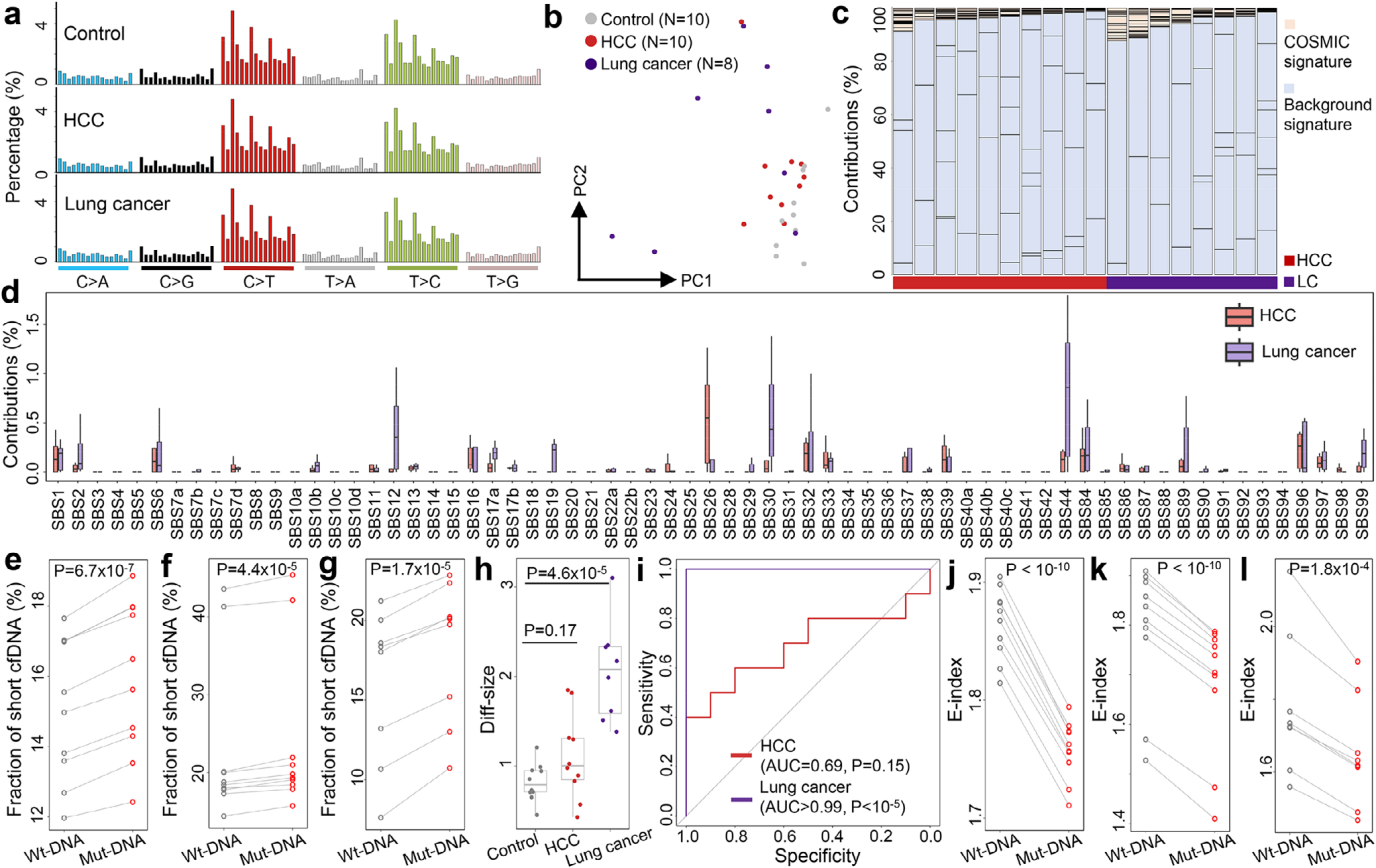
Genomic and fragmentomic features of somatic variants in cfDNA in Liang et al. cohort. (a) Mutation profiles of a non‐cancerous control, an HCC sample, and a lung cancer sample. (b) PCA result based on the mutation profiles. (c) Deconvolution results, and (d) contributions of COSMIC mutational signatures after deconvolution. (e–g) Fraction of short cfDNA fragments for Wt‐ and Mut‐DNA in (e) controls, (f) HCC samples, and (g) lung cancer samples. (h) Diff‐size values among controls and cancer samples. (i) ROC curves for differentiating cancer patients from controls using Diff‐size values. (j–l) E‐index values for Wt‐ and Mut‐DNA in (j) controls, (k) HCC samples, and l) lung cancer samples. In (e,f,g,j,k,l), P‐values were calculated using paired *t*‐tests. In (h), *P*‐values were calculated using Mann–Whitney U tests. In (i), *P*‐values were calculated using *Z*‐tests. In (b,e–h,j–l), each dot represents one sample. In (d,h), boxplots represent the median, upper and lower quartiles and whiskers indicate 1.5 × IQR.

To validate our findings, we analyzed a public cfDNA dataset from Liang et al., which includes samples from 10 hepatocellular carcinoma (HCC) patients, 8 lung cancer patients, and 10 healthy controls [42]. The mutation patterns identified in this dataset were consistent with those observed in our HCC cohort, with a clear preference for C>T and T>C variants (Figure 6a and Table S2). PCA and unsupervised clustering of the mutational signatures revealed a distinct separation between control and cancer samples (Figure 6b and Figure S4). We further used all 10 control samples as background references to deconvolute the mutational signatures in the cancer samples. As shown in Figure 6c, the majority of mutational contributions in both HCC and lung cancer samples were attributed to the “background signatures”, which corroborates the findings in our HCC cohort (Figure 2d). Notably, several COSMIC mutational signatures highly represented in our HCC cohort, SBS1, SBS16, SBS26, and SBS33, also showed prominent contributions in the HCC samples of this dataset. In contrast, lung cancer samples exhibited increased contributions from SBS30 and SBS44 compared to HCC samples, while contributions from SBS26 were reduced (Figure 6d). These results suggest that cfDNA compassing somatic variants preserve tumor‐specific information across different cancer types, with distinct mutational signatures observed between HCC and lung cancer.

Next, we extracted Mut‐ and Wt‐DNA from each sample and investigated their fragmentomic characteristics. The results are summarized in Figure 6e–l and Figure S5. Briefly, Mut‐DNA was consistently shorter than Wt‐DNA across all sample groups with statistically significant differences (*P* <10^−4^ for all groups, paired *t*‐tests; Figure 6e–g). Additionally, Diff‐size values were significantly higher in lung cancer samples, enabling perfect differentiation from controls (Figure 6h). While Diff‐size also showed a similar trend in HCC samples, the difference did not reach statistical significance. Furthermore, E‐index values for Mut‐DNA were significantly lower than for Wt‐DNA across all sample groups (Figure 6j–l). Together, these results further substantiate our findings from the HCC cohort, showing that Mut‐DNA exhibits cancer‐associated fragmentomic features in both cancer patients and non‐cancerous controls.

### 2.7 Fragmentomic Features of Variants in Enzymatic Methyl Sequencing of cfDNA

**FIGURE 7:**
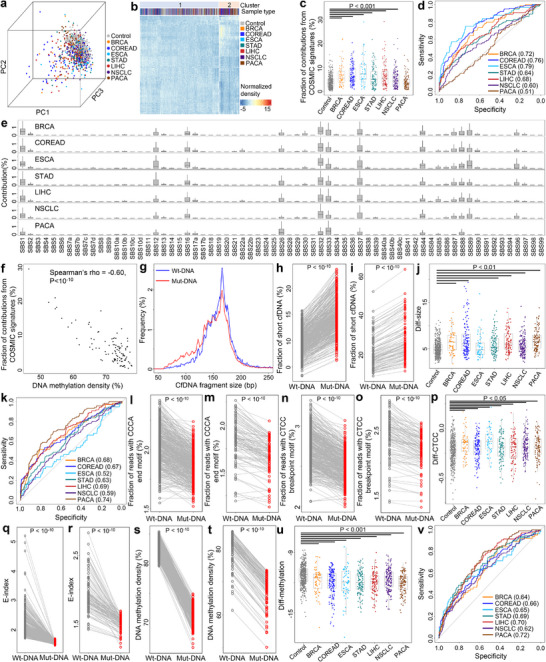
Genomic, fragmentomic, and epigenomic features of somatic variants in cfDNA in Bie et al. dataset. (a) PCA and (b) unsupervised clustering results using mutation profiles. (c) Total contributions of COSMIC mutational signatures across controls and cancer samples of different types. (d) ROC curves for differentiating cancer samples from controls using total contributions from COSMIC mutational signatures. The AUCs were recorded in parentheses. All *P* < 0.05 except PACA calculated by *Z*‐tests. (e) Contributions of COSMIC mutational signatures across different cancer types. (f) Correlation between total contributions of COSMIC mutational signatures and overall DNA methylation levels in liver cancer samples. (g) Size distributions of Wt‐ and Mut‐DNA in a liver cancer sample. (h,i) Fraction of short cfDNA fragments in Wt‐ and Mut‐DNA in (h) controls and (i) liver cancer samples. (j) Diff‐size values across controls and cancer samples. (k) ROC curves for differentiating cancer samples from controls using Diff‐size. The AUCs were recorded in parentheses. All *P* < 0.05 except ESCA calculated by *Z*‐tests. (l,m) Fraction of reads with 5’‐CCCA end motif for Wt‐ and Mut‐DNA in (l) controls and (m) liver cancer samples. (n,o) Fraction of reads with CT‐5’‐CC breakpoint motif in Wt‐ and Mut‐DNA in (n) control subjects and (o) liver cancer patients. (p) Diff‐CTCC values across controls and cancer samples. (q,r) E‐index values in Wt‐ and Mut‐DNA in (q) controls and (r) liver cancer samples. (s,t) DNA Methylation levels in Wt‐ and Mut‐DNA in (s) controls and (t) liver cancer samples. (u) Differences in methylation levels (Diff‐methylation) between Mut‐ and Wt‐DNA across controls and cancer samples for CpGs within gene bodies, and (v) corresponding ROC curves for differentiating cancer samples and controls. AUCs were recorded in parentheses, and all *P* < 0.001 calculated by *Z*‐tests. In (h,i,l–o,q–t), P‐values were calculated using paired *t*‐tests. In (c,j,p,u), P‐values were calculated using Mann–Whitney U tests. In d,k,v, P‐values were calculated using *Z*‐tests. In (a,c,f,h–j,l–u), each dot represents one sample. In (c,e,j,p,u), boxplots represent the median, upper and lower quartiles and whiskers indicate 1.5× IQR. BRCA: breast cancer, COREAD: colon/rectal cancer, ESCA: esophageal cancer, STAD: gastric cancer, LIHC: liver cancer, NSCLC: non‐small cell lung cancer, PACA: pancreatic cancer.

Considering that DNA methylation in cfDNA has been extensively studied in cancer liquid biopsy, to explore whether we could profile somatic variants and fragmentomics in such data, a pan‐cancer EM‐seq dataset from Bie et al. was collected and analyzed [43]. After quality control, we retained 1,185 cfDNA samples, including 448 non‐cancerous controls and 737 cancer samples from seven types: 64 breast cancer (BRCA), 135 colon/rectal cancer (COREAD), 56 esophageal cancer (ESCA), 109 gastric cancer (STAD), 107 liver cancer (LIHC), 150 non‐small cell lung cancer (NSCLC), and 116 pancreatic cancer (PACA) for further analysis. First, we identified somatic variants and mutation profiles across all samples. Overall, the mutation profiles were similar to those observed in whole genome sequencing (Figure S6 and Table S3). PCA of the mutation profiles revealed that control samples clustered together, while cancer samples exhibited substantial heterogeneity (Figure 7a). Unsupervised clustering identified 2 major clusters with consistent trend: 412 out of 448 controls (92.0%) were in Cluster‐1, significantly higher than random assignment (P = 3.3 × 10^−4^, binomial test). Moreover, 56.5% samples in Cluster‐1 were cancerous, while cancerous samples accounted for 84.9% in Cluster‐2 (*P* < 10^−10^, Chi‐Squared test; Figure 7b), suggesting distinct mutation profiles between cancer and control samples.

Next, we randomly selected 10 control samples as background references and deconvoluted the mutation profiles of the remaining samples against 67 COSMIC mutational signatures and the background signatures. For all samples, most mutational contributions were attributed to the “background signatures”; however, the contributions from COSMIC mutational signatures were significantly higher in cancer samples from six cancer types compared to controls (all P <0.001, except PACA, Mann–Whitney U tests; Figure 7c). ROC analysis revealed AUC values ranging from 0.60 to 0.79 for differentiating these six cancer types from controls (all P < 0.001, *Z*‐tests; Figure 7d). Common pan‐cancer mutational signatures, such as SBS1, SBS12, SBS16, SBS32, SBS33, and SBS37, were shared across cancer types, while cancer‐specific signatures, such as SBS85 and SBS90, were also identified (Figure 7e). Moreover, the total contributions of COSMIC mutational signatures were negatively correlated with overall DNA methylation levels in all cancer types (all P <10^−4^, Spearman correlation; Figure 7f; Figure S7). As tumor‐derived cfDNA molecules are hypomethylated [7], which leads to a negative correlation between overall DNA methylation levels and tumor DNA fractions in cfDNA data [44], the results suggest a positive correlation between the total contributions of COSMIC mutational signatures and tumor DNA fractions, echoing the results in our HCC cohort (Figure S2b).

We next examined the fragmentomic features associated with somatic variants by extracting Mut‐DNA and Wt‐DNA from each sample. As shown in Figure 7g–i and Figure S8, Mut‐DNA was again significantly shorter than Wt‐DNA in both cancer and control samples (*P* <10^−10^ for all groups, paired *t*‐tests). Diff‐size values were significantly higher in six cancer types compared to controls (all *P* <0.01, except ESCA, Mann–Whitney U tests; Figure 7j), with AUC values ranging from 0.59 to 0.74 for differentiating these cancer samples from control samples (all *P* < 0.01, *Z*‐tests; Figure 7k). Additionally, the use of 5’‐CCCA end motif and CT‐5’‐CC breakpoint motif were significantly reduced in Mut‐DNA compared to Wt‐DNA in both control and cancer samples (all *P* <10^−10^, paired *t*‐tests; Figure 7l–o ; Figure S9). Notably, Diff‐CTCC values were significantly elevated in all cancer types compared to controls (all *P* <0.05, Mann–Whitney U tests; Figure 7p), while Diff‐CCCA values did not differ significantly in most cancer types (Figure S9g). Furthermore, Mut‐DNA exhibited significantly lower E‐index values than Wt‐DNA across all groups (all *P* <10^−10^, paired *t*‐tests; Figure 7q,r; Figure S10), while no significant differences were observed in the fraction of reads located within nucleosomes between Mut‐DNA and Wt‐DNA (Figure S10). Together, the results confirm the aberrant fragmentomic features associated with somatic mutations in cfDNA, consistent with findings from whole genome sequencing datasets.

As the dataset was generated using EM‐seq protocol, we also analyzed DNA methylation profiles associated with somatic variants. Considering that DNA methylation is context‐dependent [45], we narrowed the analysis to CpG sites covered by both Mut‐ and Wt‐DNA; in addition, we divided the CpG sites into three groups based on their genomic contexts: promoters (defined as ± 500 bp around transcription start sites), gene bodies, and intergenic regions. As a result, in all samples (including controls), Mut‐DNA exhibited significantly lower DNA methylation levels than Wt‐DNA for all three groups of CpGs (*P* < 10^−8^ for all groups, paired t‐tests; Figure 7s,t; Figures S11 and S12). We then quantified the differences in methylation levels between Mut‐ and Wt‐DNA for each sample, referred to as “Diff‐methylation”. As shown in Figure 7u, Diff‐methylation values for CpGs within gene bodies were significantly lower in all seven cancer types compared to controls (all *P* <0.001, Mann–Whitney U tests; Figure 7u; Figure S11); ROC analysis demonstrated that Diff‐methylation values for CpGs within gene bodies could differentiate cancer samples from controls with AUCs ranging from 0.62 to 0.72 (all *P* <0.001, *Z*‐tests; Figure 7v). In contrast, Diff‐methylation values for CpGs in promoters did not show significant differences between cancer samples and controls, and Diff‐methylation values for CpGs in intergenic regions were significantly lower in three cancer samples compared to controls (Figure S12). These findings suggest that, in addition to fragmentomic features, epigenomic alterations also existed in cfDNA harboring somatic variants.

### 2.8 Artificial Intelligence‐Empowered Cancer Diagnostic Models

**FIGURE 8:**
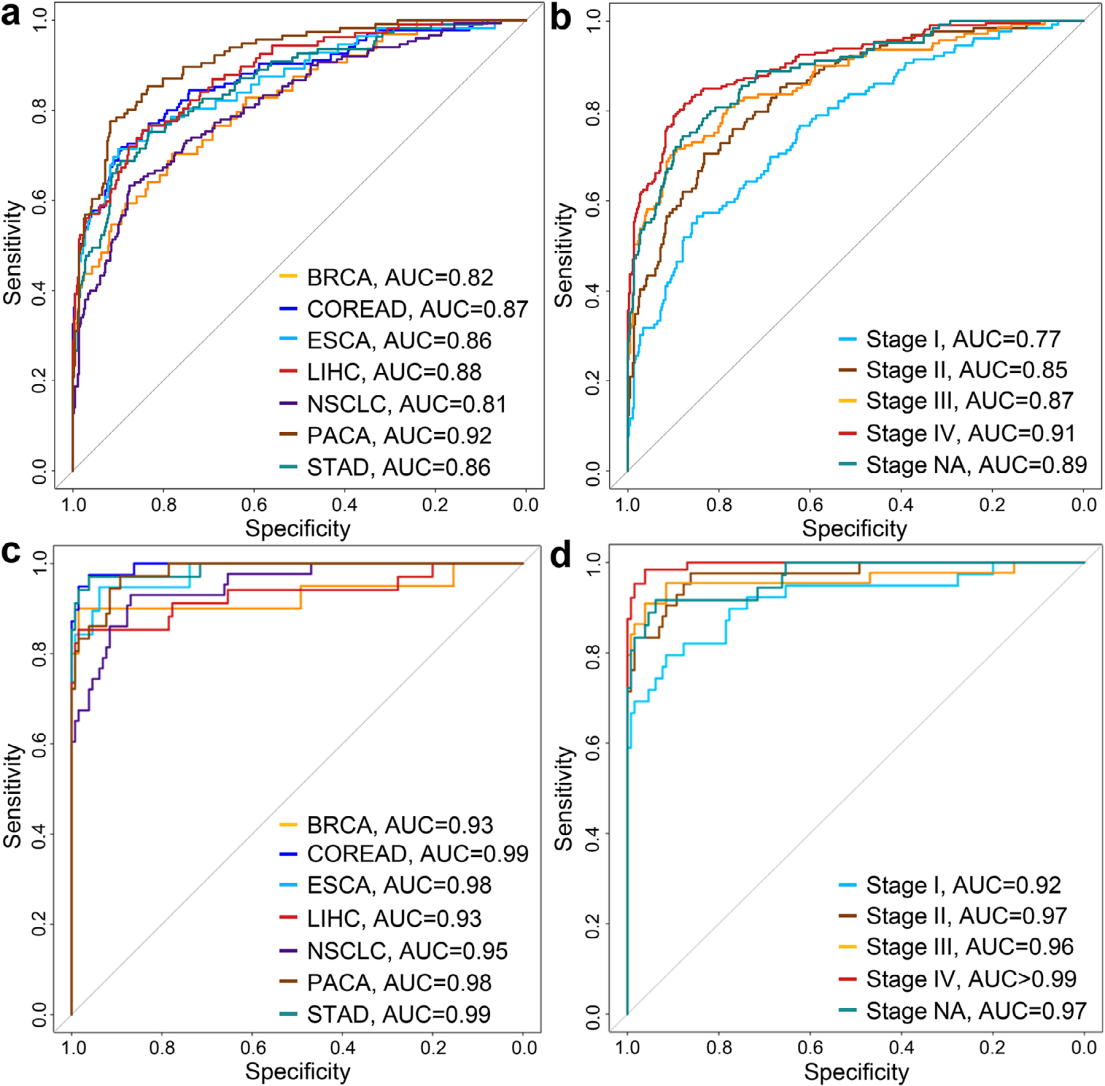
Performance of FreeSV and FreeSV+ models for cancer diagnoses. (a,b) ROC curves of FreeSV model leveraging only somatic variants‐associated features across (a) cancer types and (b) stages. (c,d) ROC curves of FreeSV+ model leveraging both somatic variants‐associated features and genomewide features on samples in the testing group across (c) cancer types and (d) stages. *P* < 10^−10^ for all AUCs, *Z*‐tests.

Building on the findings presented in Figure 7, we applied artificial intelligence (AI) to integrate genomic, fragmentomic, and epigenomic characteristics associated with somatic variants in cfDNA to develop cancer diagnostic models on the Bie et al. dataset. The features included mutation profiles, Diff‐size, Diff‐CCCA, Diff‐CTCC, Diff‐nucleosome, and Diff‐methylation for CpGs in gene bodies, totaling 101 features. We employed a gradient boosting machine (GBM) model and conducted 10‐fold nested cross‐validation repeated 100 times, using the averaged prediction scores to assess the cancerous status of the samples, as validated in previous studies [12, 46]. We termed this approach FreeSV (Fragmentomic and Epigenomic Examination of Somatic Variants in cfDNA). FreeSV demonstrated AUC values ranging from 0.81 to 0.92 across the seven cancer types, and 0.77 to 0.91 among different stages (all *P* < 10^−10^, *Z*‐tests; Figure 8a,b; Figures S13 and S14).

In practice, multiple features could be integrated toward building high‐performance diagnostic models. For instance, in Bie et al. study, the authors incorporated four genomewide features (i.e., DNA methylation, fragment size, end motif, and copy number) and built a highly accurate model named THEMIS [43]. As a proof‐of‐concept, we combined our somatic variant‐associated features with these four genomewide features (Table S3) and built a new model termed “FreeSV+” to explore whether integration of our features could further improve the performance. Of note, in this dataset, the samples were separated into non‐overlapping training and testing groups, and we built the FreeSV+ model using the samples in the training group only, then directly applied the model on the testing group. On the training group, we measured the performance of FreeSV+ using 10‐fold nested cross‐validation repeated 100 times, which resulted in ROC curves with AUCs ranging from 0.96 to 0.99, all of which were higher than THEMIS (Figures S15 and S16). On the testing group, the AUCs were comparable between FreeSV+ and THEMIS (Figure 8c,d); however, at 99% specificity, FreeSV+ correctly identified 183 out of 225 cancer samples (sensitivity = 81.3%, 95% CI: 75.6%–86.2%; Table 1), which was significantly higher than THEMIS's 74.4% (P = 0.0076, binomial test); moreover, FreeSV+ correctly identified 71.6% stage I/II cancer samples, which was also significantly higher than THEMIS's 61.0% (P = 0.037, binomial test). Together, these results highlight the feasibility of integrating multi‐omics features toward high‐performance diagnostic models, as well as the translational significance of our somatic variant‐associated features.

**TABLE 1:** Performance of FreeSV+ model on samples in the testing group in Bie et al. dataset.

		Individualsanalyzed	99% specificity		
			Individuals detected	Sensitivity (%)	95% CI (%)
Healthy		130	1	—	—
Cancer		225	183	81.3	75.6‐86.2
Type					
	BRCA	20	16	80.0	56.3‐94.3
	COREAD	39	35	89.7	75.8‐97.1
	ESCA	19	16	84.2	60.4‐96.6
	LIHC	34	28	82.4	65.5‐93.2
	NSCLC	43	28	65.1	49.1‐79.0
	PACA	36	29	80.6	64.0‐91.8
	STAD	34	31	91.2	76.3‐98.1
Stage					
	I	39	26	66.7	49.8‐80.9
	II	42	32	76.2	60.5‐87.9
	III	44	37	84.1	69.9‐93.4
	IV	64	59	92.2	82.7‐97.4
	NA	36	29	80.6	64.0‐91.8

## 3 Discussion

In this study, through integrative and comprehensive analysis on fragmentomic characteristics of somatic variants in cfDNA, we showed that the somatic variants, even in low‐pass data, are not stochastic while preserve informative characteristics associated with their cells‐of‐origin (i.e., clonal hematopoiesis or tumor) and possess translational potential. Over the past decade, research focus on cancer liquid biopsy has partially shifted from genomic and epigenomic to fragmentomic characteristics of cfDNA [8, 14, 16], which hold the advantage of working with shallow sequencing data. In such low‐pass cfDNA data, it is highly challenging to precisely differentiate clonal hematopoiesis‐ and tumor‐derived somatic variants from technical artefacts. Hence, the aim of this study is not to determine the origin of each somatic variant in low‐pass cfDNA data, while to classify whether tumor‐associated signals exist or not in the somatic variants. Interestingly, principal component analysis (PCA) and unsupervised clustering analyses both showed certain degree of separation between control and cancer samples (Figure 2, 6, 7), suggesting the feasibility of discern tumor‐associated signals from the somatic variants in low‐pass cfDNA data from cancer patients. Overall, our findings on the genomic profiles of somatic variants in cfDNA are highly consistent with previous work by Wan et al. [29], while our approach differs from that of Wan et al. in several key aspects. The primary difference is our use of a control panel to evaluate the background noises in addition to the COSMIC mutational signatures during mutation profile deconvolutions. As shown in Figure 2, 6, 7, after deconvolution, the major contributions were attributed to the “background signatures”; importantly, cancer samples showed higher contributions from COSMIC mutational signatures. The total contributions of COSMIC mutational signatures were positively correlated with tumor DNA fractions in cfDNA (Figure 7f; Figures S2b and S7). These results suggested that our approach can effectively filter out noises, which are often protocol/platform‐dependent [47], and emphasize the cancer‐associated signals. Moreover, our approach was validated in both whole genome sequencing and EM‐seq datasets, suggesting the robustness and generality of our approach.

Beyond genomic analysis, we conducted a comprehensive investigation on the fragmentomic characteristics in cfDNA associated with somatic variants, an area not previously explored in detail. Surprisingly, even in non‐cancerous controls, cfDNA molecules covering somatic variants (“Mut‐DNA”) exhibited cancer‐like fragmentomic patterns compared to those covering reference alleles (“Wt‐DNA”). These patterns included shorter fragment sizes, aberrant end or breakpoint motif usage, and higher proportions of ends located within nucleosomes. As shown in Figure 5, cfDNA fragments harboring clonal hematopoiesis‐derived variants exhibit such cancer‐like hallmark features, underscoring the pervasive influence of clonal hematopoiesis signals on cfDNA fragmentomics. In a prior study, we demonstrated that DNA methylation plays a key role in cfDNA fragmentation, with lower methylation levels correlating with shorter cfDNA fragments [1]. Previous studies have also shown that mutations in DNMT3A and TET2, which occur during clonal hematopoiesis [48, 49], lead to decreased DNA methylation in aged hematopoietic cells [50, 51]. Accordingly, Mut‐DNA in the EM‐seq data exhibited decreased DNA methylation levels (Figure 7s,t), suggesting that in non‐cancerous subjects, the age‐related clonal hematopoiesis could be a major contributor of somatic variants in cfDNA and serves as the cause of cancer‐like fragmentomic patterns in Mut‐DNA. The findings thus provided evidences to elucidate the shortness of cfDNA molecules observed in aged people [1, 52] and highlighted the necessity of age‐matched controls in cancer diagnosis [53]. Moreover, in cancer cfDNA samples, size patterns had been utilized as a valuable filter for the identification of tumor‐derived mutations [19, 54]; however, our findings suggested that such filter could not eliminate the clonal hematopoiesis‐related variants as they are also associated with shorter cfDNA, which partially explained the false positives in previous studies [19, 54]. In clinical, clonal hematopoiesis is associated with worse clinical outcome in various diseases, including cardiovascular diseases and cancer [55, 56, 57]; hence, our findings suggested the possibility of investigating clonal hematopoiesis by incorporating mutational profiles and fragmentomics in cfDNA [39]. Besides clonal hematopoiesis, a recent study reported cancer‐like fragmentomic patterns in cfDNA from non‐cancerous patients with TP53 mutations. In these cases, mutant TP53 altered chromatin accessibility, contributing to the observed fragmentomic changes [58]. Besides human data, somatic variant‐associated cancer‐like fragmentomic patterns was validated in murine cfDNA samples (Figure 4). Together, the results indicated that investigating cfDNA fragments covering somatic variants in non‐cancerous subjects offers novel insights into the regulation of cfDNA fragmentomics, shedding light on the mechanisms underlying cfDNA fragmentation and its implications for cancer diagnostics.

In addition, we defined several parameters to quantitatively assess the fragmentomic and epigenomic alterations between Mut‐ and Wt‐DNA, including Diff‐size, Diff‐CCCA, Diff‐CTCC, Diff‐nucleosome, and Diff‐methylation. Notably, in most analyses, these parameters revealed significant differences between cancer patients and controls. For example, across all datasets analyzed, Diff‐size values were consistently higher in cancer patients than in controls, in line with the fact that tumor‐derived cfDNA molecules are shorter than non‐tumor‐derived ones [1, 9]. When examining breakpoint motif usage, Diff‐CTCC values were lower in cancer patients in the HCC cohort, consistent with prior studies reporting reduced usage of the CT‐5’‐CC breakpoint motif in cfDNA from cancer patients [11]. Interestingly, in Bie et al. EM‐seq dataset, Diff‐CTCC values were elevated in cancer patients (Figure 7p), which may be attributed to high biases in EM‐seq data caused by cytosine‐to‐thymine conversions [59]. Overall, these somatic variant‐associated parameters showed consistent differences between cancer patients and controls as demonstrated in various pan‐cancer datasets (Figures 3, 6 and 7), suggesting that they hold the promise as a novel category of biomarkers for cancer diagnosis. Although the performances of these parameters were limited when used alone, diagnostic models that integrated them using AI showed favorable performances. For example, our FreeSV model was validated using more than 1100 samples, and its performance demonstrated the translational merit of somatic variant analysis and the innovative features proposed in this study, particularly when applied to low‐pass cfDNA data. Moreover, as a proof‐of‐concept, we further integrated these somatic variant‐associated features identified in this study with genomewide features leveraged in Bie et al. study. The performances of our models were evaluated using both nested cross validation and separated testing samples. As shown in Figure 8 and Table 1 and Figures S14 and S16, the performance (especially the sensitivity) of FreeSV+ on testing group was significantly higher than using either somatic variants‐associated features or the THEMIS model with genomewide features alone. The results thus demonstrated the translational significance of integrating multi‐omics features in cfDNA toward building high‐performance cancer diagnostic models.

In summary, this study provides a comprehensive profiling of genomic and fragmentomic characteristics in low‐pass cfDNA data without paired germline genotypes. We show that cfDNA molecules harboring somatic variants in controls exhibit cancer‐like fragmentomics compared to those covering reference alleles. Additionally, our computational approach proposes a new paradigm for integrative genomic, fragmentomic, and epigenomic analyses in cfDNA toward high‐accuracy cancer diagnostic models.

## 4 Methods

### 4.1 Ethics Approval and Sample Processing

This study was approved by the Ethics Committee of Shenzhen Bay Laboratory and the Ethics Committee Shenzhen Third People's Hospital. Hepatocellular carcinoma (N = 1) and colorectal cancer (N = 1) patients were recruited in Shenzhen Third People's Hospital with consents. For each subject, 10 mL peripheral blood was collected using EDTA‐containing tubes, stored at 4 °C and processed within 4 h. Tumor samples were collected during surgical resections and the specimens were immediately washed using physiological saline and stored in liquid nitrogen until usage. Animal study was conducted according to protocols approved by SZBL Animal Center (approval No.: AESK202302). Wildtype C57BL/6J strain mice (N = 12) were housed under specific pathogen‐free conditions with a 12h light/dark cycle, at a temperature of 20–26°C, and a relative humidity of 40–70%; mice were fed a standard mouse chow diet and were sacrificed between 12–30 weeks. For both human subjects and mice, blood samples were centrifuged at 1600 g, 4°C for 15 min, and the plasma and white blood cells portions were harvested; the plasma portion was further re‐recentrifuged at 16 000 g, 4°C for 15 min to remove retaining blood cells [1, 60]; plasma samples were stored at −80 °C until further usage. CfDNA extraction and library preparation were performed as in our previous study [12]. Briefly, for each sample, cfDNA was extracted from 300–600 µL plasma with MagPure Circulating DNA LQ Kit (Magen, #IVD5432) and 3–6 ng cfDNA was used to construct libraries using VAHTS Universal DNA Library Prep Kit for MGI (Vazyme Biotech Co.,Ltd, #NDM607) following the manufacturers’ instructions. For genotyping of PBMCs and tumors, genomic DNA extraction and library preparation were performed as in our previous study [12]. Briefly, for each sample, genomic DNA was extracted with DNeasy Blood & Tissue Kit (QIAGEN, #69506) and sonicated to 200–500 bp with Covaris S220 (Covaris) following manufacturer's instructions; 100–300 ng sonicated genomic DNA were used to construct libraries using VAHTS Universal DNA Library Prep Kit for MGI (Vazyme Biotech Co.,Ltd, #NDM607) following manufacturer's instructions. Both CfDNA and genomic DNA libraries were sequenced on an MGISEQ‐T7 (MGI) sequencer in paired‐end 100 bp mode.

### 4.2 CfDNA Sequencing Data Processing

For cfDNA whole‐genome sequencing data, raw reads were first preprocessed using Ktrim software (version 1.5.0) [61] to remove sequencing adapters and low‐quality cycles. The preprocessed reads were then aligned to reference human genome (NCBI GRCh38) or mouse genome of C57BL/6J strain “B6Eve” (Jackson Laboratory) [38] for human and mouse data, respectively, using BWA‐MEM software (version 0.7.17) [62]. Alignment results were sorted and indexed using SAMtools (version 1.17) [63]. PCR duplicates were identified and removed using the Picard package (version 2.23.4). EM‐seq data was analyzed using Msuite2 software (version 2.1.0) [64, 65], an all‐in‐one pipeline containing quality control, read alignment, and methylation calling. Prior to alignment, the tailing 25 bp in read 1 and leading 25 bp in read 2 were trimmed to avoid overhang issues inherent to cfDNA [66]; hisat2 software (version 2.2.1) [67] was employed as the underlying aligner and all the other parameters were kept default. EM‐seq samples with an overall mapping rate below 80%, a cytosine‐to‐thymine conversion rate below 98%, fewer than 40 million reportable alignments, or abnormal methylation levels in promoter regions were excluded from downstream analysis. For all datasets, samples with more than 100 million reads were randomly down‐sampled to 60 million reads for downstream analyses. For hepatocellular carcinoma (HCC) samples, tumor DNA fractions were calculated using ichorCNA algorithm (version 0.3.2) [68] with a window size of 500 kb, as described in our previous study [12].

### 4.3 Identification of Somatic Variants in cfDNA

For both whole genome sequencing and EM‐seq data, unmapped reads and secondary alignments were removed. Variant calling was then performed using the “mpileup” function in BCFtools software (version 1.12) [69] with “‐q 60” option to exclude alignments with a mapping quality lower than 60 and “‐Q 30” option to exclude bases with a sequencing quality score below 30. The pileup sequences were subsequently processed with the “call” function and “‐c” option in BCFtools to identify candidate variants. For EM‐seq data, due to C‐to‐T conversion during library preparation, cytosines in the reference genome were treated differently: thymines in the sequencing data were not considered as candidate variants. Variants involving insertions/deletions, those on sex chromosomes or mitochondria, loci with more than one non‐reference allele, with a detection quality below 30, or continuous variants were all discarded.

For human samples, variants overlapping with genomic regions defined as analytically problematic by the ENCODE project [70], as well as variants within the top 1% of coverage, were also discarded. We then filtered out variants annotated in dbSNP database (version 156) [71] with an allele frequency greater than 0.1%. As all samples analyzed in this study were from east‐Asian patients, variants with an allele frequency greater than 0.1% in the Nyuwa [72], ChinaMAP [73], Korea1K [74], or ToMMo [75] databases were also removed. Cancer‐associated mutations from the COSMIC database (version 99) were retained for further analyses. For the Bie et al. dataset, one sample (accession ID: HRR1236003) was excluded due to an abnormally high number of variants. For murine cfDNA samples, variants within the top 10% of coverage were filtered out. The remaining variants, all of which were substitutions on autosomes, were retained for downstream analyses.

### 4.4 Mutation Profiles and Deconvolution Analysis

The “MutationalPatterns” package (version 3.12) [76] was used to calculate and normalize the frequencies of 96 mutational profiles for each sample. The normalized mutation profiles were then subjected to principal component analysis (PCA) and unsupervised clustering using the “ComplexHeatmap” package (version 2.18) [77]. The 86 single‐base substitution mutational signatures were downloaded from the COSMIC database (release v3.4). Signatures annotated as sequencing artifacts (N = 19) were excluded, leaving 67 signatures for deconvolution.

To infer tumor‐associated mutational signatures in cfDNA, a panel of control subjects (N = 8, 10, and 10 for our HCC cohort, Liang et al., and Bie et al. datasets, respectively) was randomly selected to simulate the background noises in cfDNA, which includes artifacts arising from experimental or sequencing errors and clonal hematopoiesis. The mutation profiles of these controls were combined with those in COSMIC to form a reference pool for deconvolution. The non‐negative matrix factorization algorithm, implemented in the “NMF” package (version 0.27) [78], was then applied to deconvolute the mutational profiles of the remaining samples. For each cfDNA sample, the predicted contributions from the COSMIC signatures were considered “tumor‐associated” and used for downstream analyses.

### 4.5 Fragmentomic and Epigenomic Features in cfDNA

For each cfDNA sample, reads overlapping somatic variant loci identified within the sample were extracted. Reads that covered multiple variants were excluded. For all variant loci, reads carrying the reference alleles were classified as “Wt‐DNA”, while those compassing variant alleles were classified as “Mut‐DNA”. Fragmentomic features were then calculated separately for the Wt‐DNA and Mut‐DNA reads for all samples.

CfDNA molecules shorter than or equal to 150 bp (i.e., ≤150 bp) were considered as short fragments, and the fraction of short fragments was calculated as a size feature for each sample. For motif analysis, as described in our previous studies [10, 79], 4‐mer sequences at the 5’ end of cfDNA fragments were extracted, and the frequency of each 4‐mer sequence was calculated. The fraction of CCCA sequences was used to assess the 5’‐CCCA end motif usage. Additionally, 4‐mer sequences using 2 bases upstream and 2 bases downstream of the 5’ end were calculated, with the fraction of CTCC sequences used to assess the CT‐5’‐CC breakpoint motif usage [34]. Motif diversity scores were calculated as in our previous studies [10, 79] and were normalized to 0–1 where 1 stands for the highest randomness.

E‐index values and the fraction of fragment ends located within nucleosomes were calculated as previously described [1, 12]. Briefly, cfDNA ends were analyzed in an orientation‐aware manner [37]: for each cfDNA molecule, its fragment ends with lower and higher genome coordinates were termed as upstream (U) and downstream (D) end, respectively, and processed separately in downstream analyses. To calculate E‐index, cfDNA reads from a pool of healthy controls were collected to build a model of end distribution, and the consistency of cfDNA ends in the sample‐of‐interest with this model was calculated using a weighted average approach (referred to as the E‐index) as illustrated in the following formula:

$$E-\mathrm{index}\;=\frac{1}{N}\;\sum_{i}M_{U}+M_{D}$$

where *N* denoted the read number of the working sample (e.g., Wt‐DNA), *i* denoted each sequencing read, *M_U_*, and *M_D_* denoted the counts of its ending positions serving as *U* and *D* ends in the model, respectively.

For each cfDNA end, the nearest nucleosome annotated in the GM12878 cell line (lymphoid lineage) was identified [12, 35]. The distance between the cfDNA end and the nucleosome center was calculated, with negative distances assigned when the cfDNA end was upstream of the nucleosome center. The fraction of cfDNA fragment ends located within ± 50 bp of nucleosome centers was calculated using the following formula [1, 12, 37]:

$$\begin{array}{ccc} & & Fraction\;of\;ends\;within\;nucleosomes\\ & & =\frac{No.\;of\;ends\;within\;\pm 50\;bp\;of\;nucleosome\;centers\;}{Total\;end\;count}\;\times 100\% \end{array}$$

For DNA methylation analysis, only variant loci with reads covering both the reference and variant alleles were retained for comparison of the same set of CpGs. DNA methylation levels for Wt‐ and Mut‐DNA were separately called using the “meth.caller.CpG” and “pair.CpG” programs in Msuite2 software with default parameters. For each sample, the CpG sites were divided into three groups: promoters (defined as ± 500 bp around transcription start sites), gene bodies, and intergenic regions; then DNA methylation densities were calculated for these three groups separately. DNA methylation density was calculated as the fraction of Cytosines at CpG sites in the sequencing data as shown in the following formula:

$$\begin{array}{ccc} & & DNA\;methylation\;density\\ & & =\frac{No.\;of\;Cytosines\;}{No.\;of\;Cytosines+No.\;of\;Tymines}\;\;\times 100\% \end{array}$$

where Cytosines indicated methylated CpG while Thymies indicated unmethylated CpG in EM‐seq protocol.

### 4.6 Identification of Tumor‐and Clonal Hematopoiesis‐Derived Somatic Variants

For hepatocellular carcinoma, colorectal cancer, and breast cancer samples with paired PBMC and tumor data, raw sequencing data were processed following the guidelines in Genome Analysis Toolkit (GATK; v4.2.3.0) [80], including read alignment, duplicate marking, and base quality score recalibration. Genomic loci with ≥30‐fold coverage and with mapping quality and base quality ≥20 in both tumor and paired PBMC data were retained for downstream analysis. To identify clonal hematopoiesis‐derived somatic variants, we examined all heterozygous loci in PBMCs and selected sites where: (i) the minor allele was supported by ≥3 reads in PBMCs, and (ii) the corresponding locus was homozygous in tumor. For such loci, the minor alleles were called as clonal hematopoiesis‐derived somatic variants, while major alleles were assigned as germline. Similarly, loci with heterozygous genotypes in tumor tissues while homozygous in the matched PBMCs were called as tumor‐derived somatic variants. For the Nix et al. dataset which only genotyped the PBMCs, loci with ≥300‐fold coverage, mapping quality ≥20 and base quality ≥20, and a minor allele frequency between 2–30% were selected, following thresholds adopted in the original study [41, 81]. For these loci, the minor alleles were attributed to clonal hematopoiesis, while major alleles were considered germline. For each individual, cfDNA reads overlapping loci harboring somatic variants were subsequently extracted. To minimize potential confounding, reads spanning multiple variant sites were excluded. Based on genotypes at these loci, cfDNA fragments were categorized into distinct groups (clonal hematopoiesis‐derived, tumor‐derived, and germline), and fragmentomic features were calculated separately for each group.

### 4.7 Building and Evaluating FreeSV and FreeSV+ Models

To build FreeSV model, all the somatic‐variant associated features, including mutation profiles (N = 96), Diff‐size, Diff‐CCCA, Diff‐CTCC, Diff‐nucleosome, and Diff‐methylation for CpGs in gene bodies (totally 101 features; Table S3) were used. A gradient boosted decision tree (GBDT) algorithm, implemented in the “gbm” package (version 2.1.9) in R software (version 4.2.0), was used to build diagnostic models using these features. Hyperparameter tuning was performed using the “caret” package (version 6.0.94). We employed a 10‐fold nested cross‐validation scheme as follows: all samples were randomly split into 10 equal‐sized subsets, with nine subsets used for training model and the remaining subset used for evaluating the performance of the model. This process was repeated for each subset, and the prediction results for each testing subset were collected. Hence, for each sample, it was only predicted by a model trained using samples without itself to eliminate information disclosure. The whole procedure was repeated 100 times, and the average prediction score was calculated and determined as prediction score for each sample. The area under the receiver operating characteristic (ROC) curve (AUC) was used to quantify model performance. The relative importance scores of each feature (extracted during model training and averaged) were shown in Figure S13.

To build FreeSV+ model, besides the features used in FreeSV, four additional genomewide features (DNA methylation, fragment size, end motif, and copy number) used in THEMIS model [43] was also leveraged (totally 105 features; Figure S13 and Table S3). For a fair comparison with THEMIS model, data separation performed in Bie et al. study was utilized: a total of 355 samples (130 controls and 225 cancer cases) were held out as the independent testing group, and the rest samples were used as the training group. The FreeSV+ model was trained on the training group using the same approach as FreeSV; the trained model was then directly applied to the testing group to examine its performance. Besides ROC analyses, a specificity of 99% (which parameter was used in Bie et al. study [43]) was employed to evaluate and compare the sensitives of FreeSV+ and THEMIS models.

### 4.8 Statistical Analysis and Reproducibility

All statistical analyses were performed using R software (version 4.2.0). Parametric tests (e.g., paired t‐test) were used to compare features in paired Mut‐ and Wt‐DNA samples, while non‐parametric tests (e.g., Mann–Whitney U test) were employed for other comparisons. In Liang et al. dataset, post‐treatment lung cancer samples (N = 2) were excluded from the analysis. In Bie et al. dataset, 92 samples were discarded during quality control. For all cfDNA data, samples with more than 100 million reads were randomly down sampled to 60 million reads for downstream analyses.

## Author Contributions

Conception, design, and study supervision: K.S.; Development of methodology: Z.Z., Y.A., K.S.; Acquisition of data: all authors; Analysis and interpretation of data: all authors; Writing the manuscript: Z.Z., Y.A., K.S.; Review and edit of the manuscript: K.S.

## Conflicts of Interest

K.S. had filed patent applications based on the method developed in this work; the remaining authors declare no conflict of interests.

## Supporting information




**Supporting File 1**: advs73772‐sup‐0001‐SuppMat.docx.

**Supporting File 2**: advs73772‐sup‐0002‐Table S1‐S3.xlsx.

## Data Availability

Murine cfDNA whole genome sequencing data had been deposited to Sequencing Read Archive (SRA) in NCBI with BioProject ID PRJNA1223612. Raw cfDNA whole genome sequencing data for the HCC cohort were retrieved from Genome Sequence Archive in National Genomics Data Center (GSA‐Human) under accession number HRA005521. Public datasets were obtained from CNGB Nucleotide Sequence Archive with accession number CNP0000680, and GSA‐Human with accession number HRA003209. Nucleosome track for GM12878 cell line was downloaded from NucMap database [82], which recorded ∼13.9 million nucleosome‐protected regions accounting for ∼90% of the human genome. Related processed files and programs/scripts had been deposited at Zenodo (https://zenodo.org/records/14849892) and github (https://github.com/hellosunking/cfDNA‐epiSNV), respectively.
