# Supplementary material for: Cancer‐Like Fragmentomic Characteristics of Somatic Variants in Cell‐Free DNA
Source: Adv Sci (Weinh). 2026 Jan 22;13(16):e14819. doi: 10.1002/advs.202514819 (PMC13042374; doi:10.1002/advs.202514819)
Supplement: Supplementary file 1 — Supporting File 1: advs73772‐sup‐0001‐SuppMat.docx. [file ADVS-13-e14819-s002.docx]

**Cancer-like fragmentomic characteristics of somatic variants in cell-free DNA**

Zhang and An et al.

This supplementary file contains Supplementary Figures S1-S16.


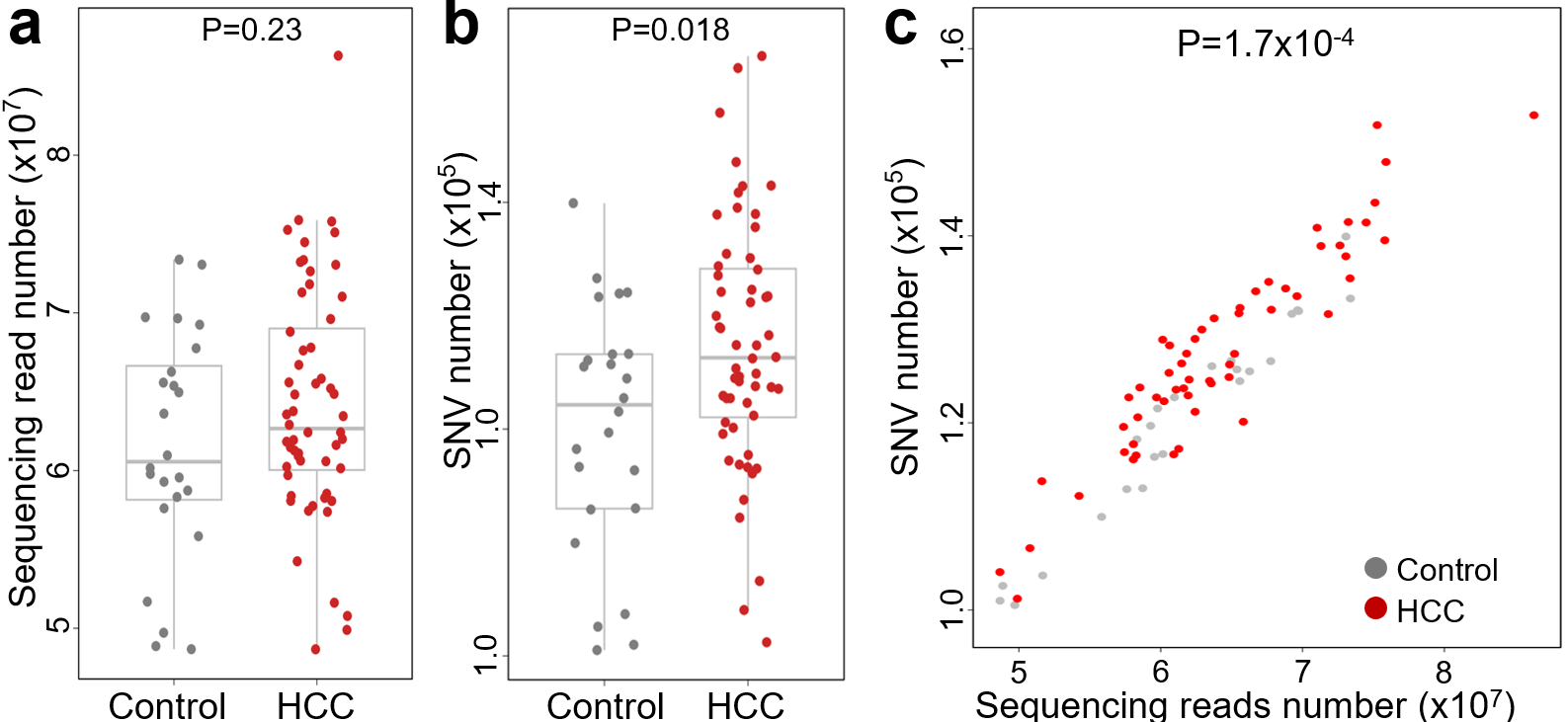


**Fig. S1. Sequencing depth and somatic variants (SNVs) in HCC cohort. a)** Sequencing read counts, and **b)** No. of somatic variants (SNVs) between controls and HCC samples. **c)** Correlation between sequencing read counts and somatic variant numbers in controls and HCC samples. Each dot represents one sample. In **a,b**, p-values were calculated using Mann-Whitney U test. In **c**, p-value was calculated using Chow test. In **a,b**, boxplots represent the median, upper and lower quartiles and whiskers indicate 1.5x IQR.


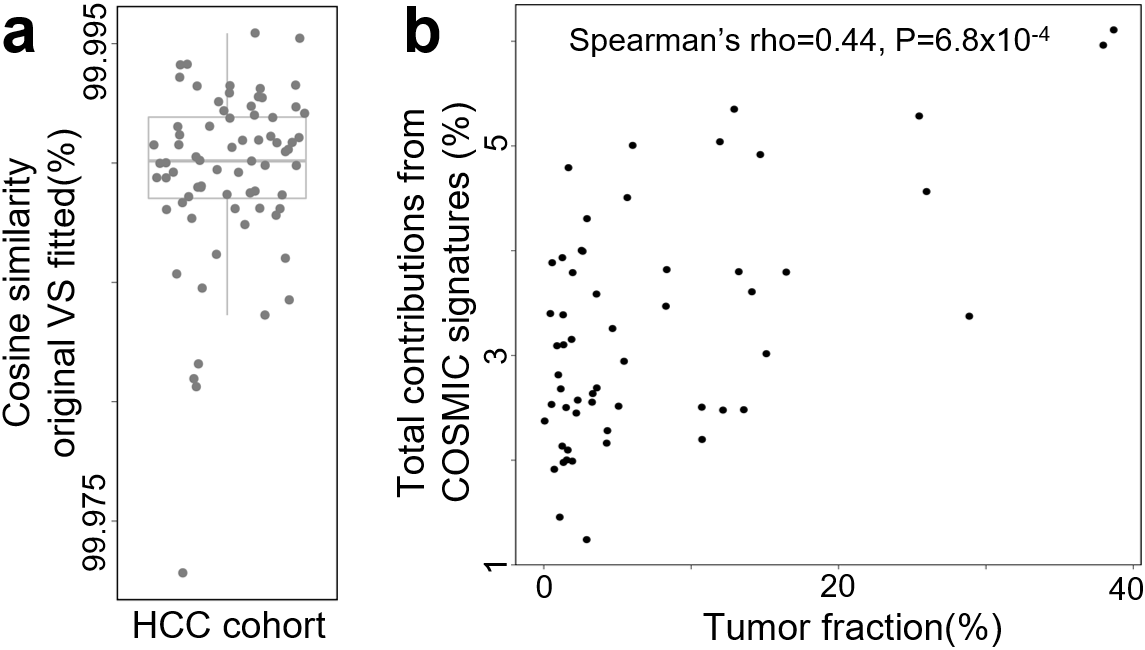


**Fig. S2. Deconvolution of mutation profiles in HCC cohort. a)** Cosine similarities between the original mutation profiles and the fitting results in all samples. **b)** Correlation between tumor fractions and total contributions from COSMIC signatures in HCC samples. Each dot represents one sample. In **a**, boxplots represent the median, upper and lower quartiles and whiskers indicate 1.5x IQR.


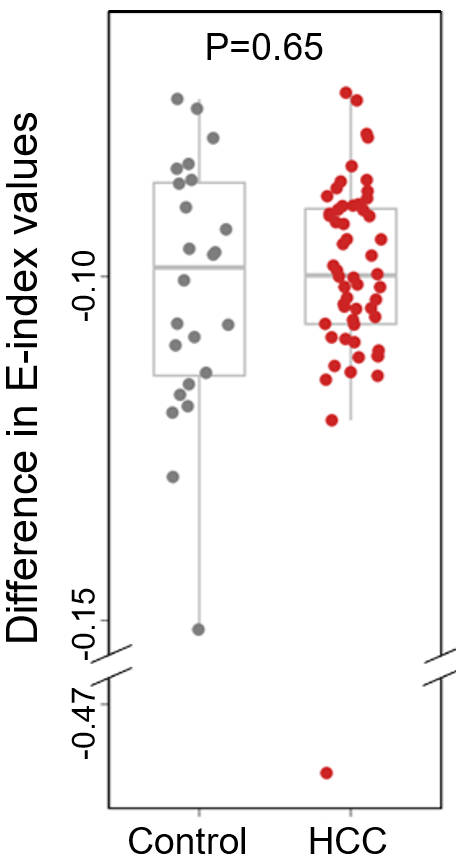


**Fig. S3. Differences in E-index values between Mut- and Wt-DNA in HCC cohort.** P-value was calculated using Mann-Whitney U test. Each dot represents one sample. Boxplots represent the median, upper and lower quartiles and whiskers indicate 1.5x IQR.


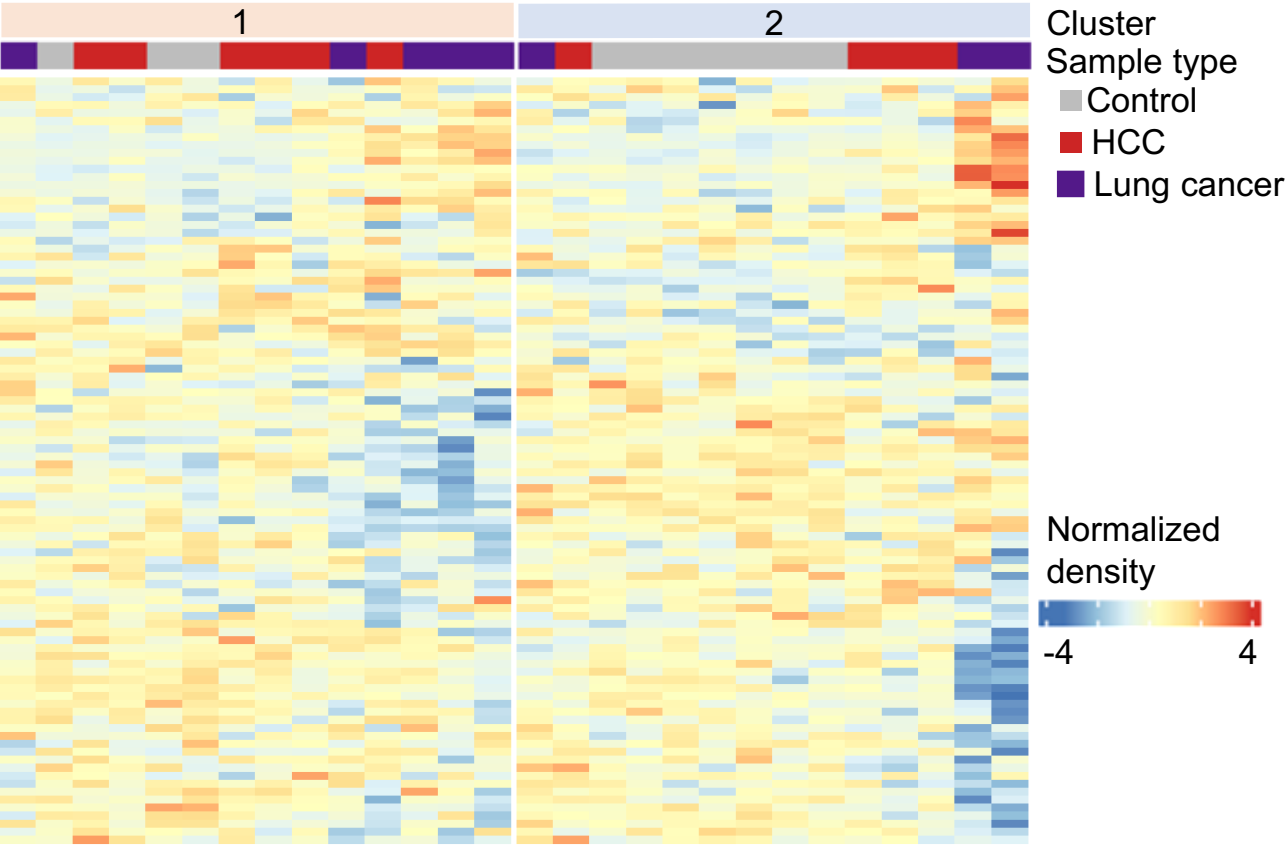


**Fig. S4. Unsupervised clustering result based on mutation profiles in Liang et al. cohort.**


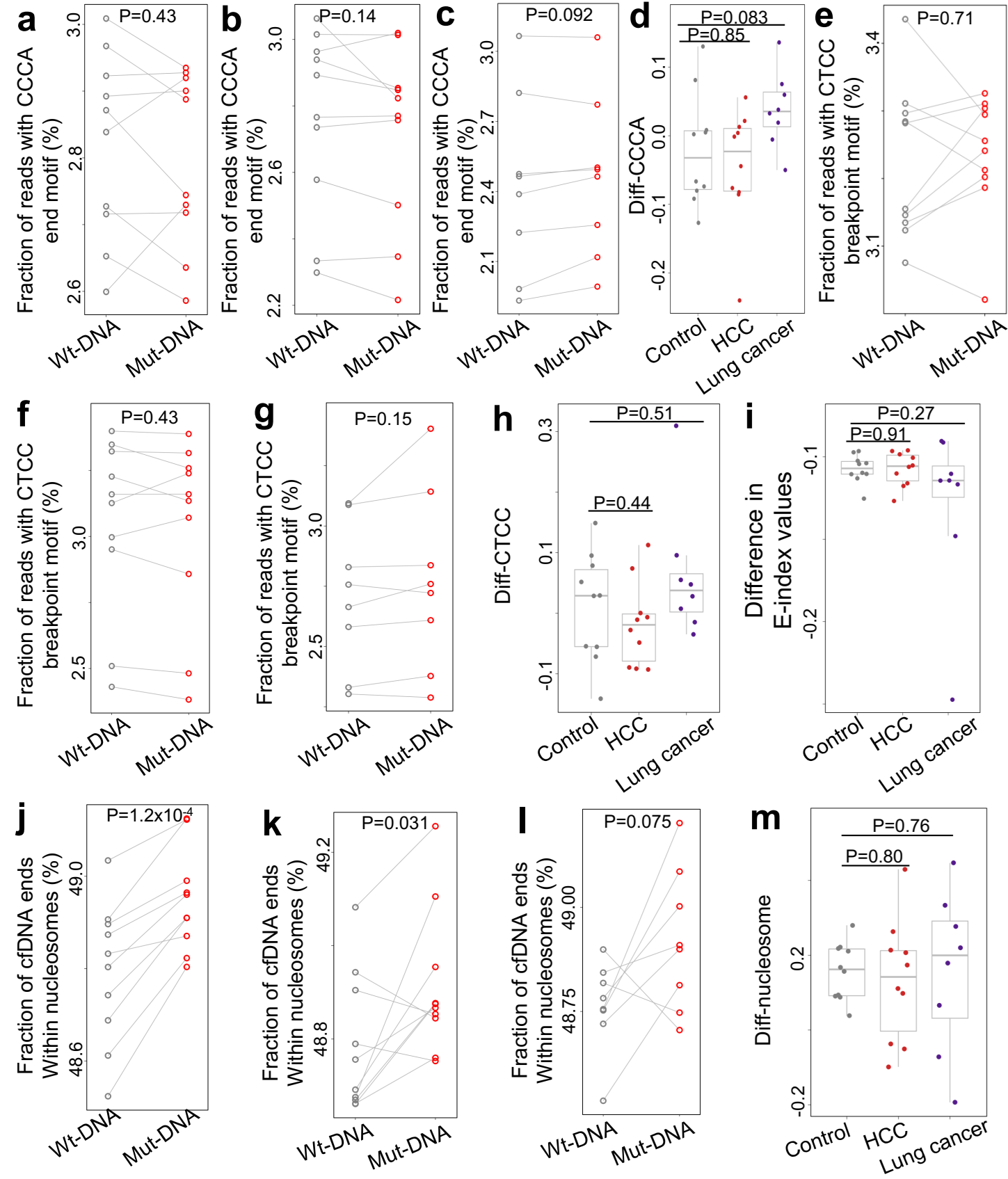


**Fig. S5. Fragmentomic features of cfDNA carrying somatic variants in Liang et al. cohort. a-c)** Fraction of reads with CCCA end motif for Wt- and Mut-DNA in **a)** controls, **b)** HCC samples, and **c)** lung cancer samples. **d)** Differences in fraction of reads with CCCA end motif (Diff-CCCA) between Mut- and Wt-DNA in controls and cancer samples. **e-g)** Fraction of reads with CTCC breakpoint motif for Wt- and Mut-DNA in **e)** controls, **f)** HCC samples, and **g)** lung cancer samples. **h)** Differences in fraction of reads with CTCC breakpoint motif (Diff-CTCC) between Mut- and Wt-DNA in controls and cancer samples. **i)** Differences in E-index between Mut- and Wt-DNA between controls and cancer samples. **j-l)** Fraction of ends located within nucleosomes for Wt- and Mut-DNA in **j)** controls, **k)** HCC, and **l)** lung cancer samples. **m)** Differences in fraction of ends located within nucleosomes (Diff-nucleosome) between Mut- and Wt-DNA in controls and cancer samples. In **a-c,e-g,j-l**, p-values were calculated using paired t-tests. In **d,h,i,m**, p-values were calculated using Mann-Whitney U test. In **a-m**, each dot represents one sample. In **d,h,i,m**, boxplots represent the median, upper and lower quartiles and whiskers indicate 1.5x IQR.


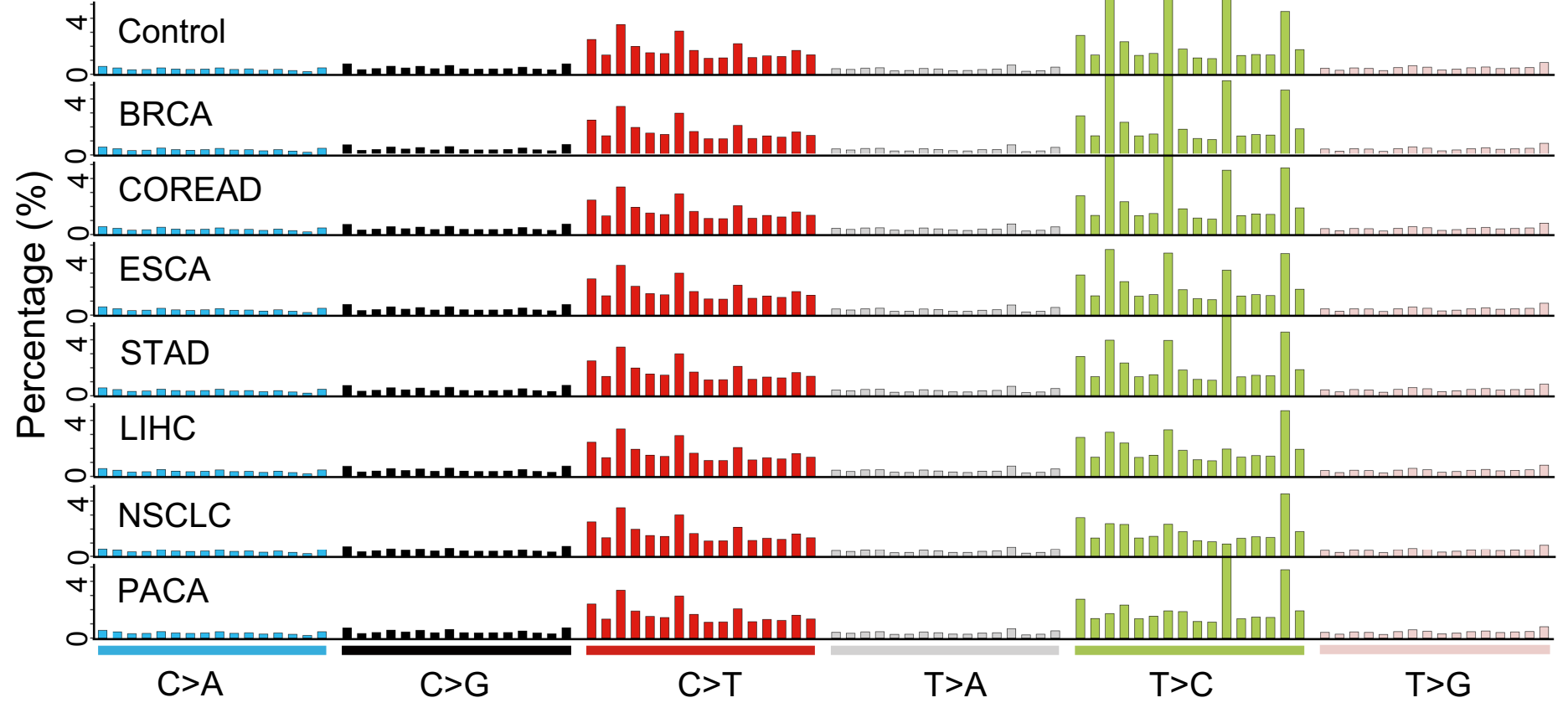


**Fig. S6. Mutation profiles in Bie et al. cohort.** BRCA: breast cancer, COREAD: colon/rectal cancer, ESCA: esophageal cancer, STAD: gastric cancer, LIHC: liver cancer, NSCLC: non-small cell lung cancer, PACA: pancreatic cancer.


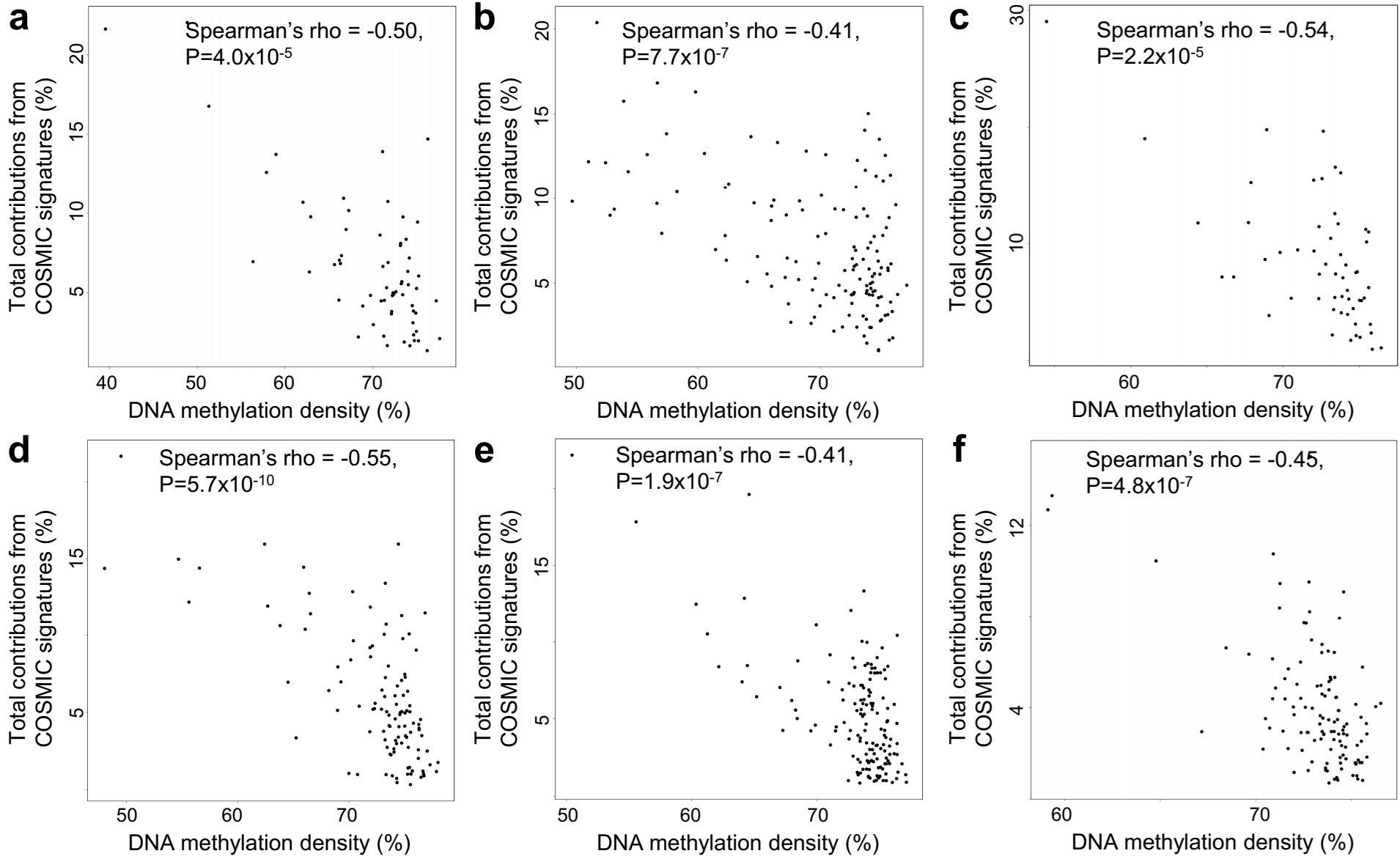


**Fig. S7. Correlation between DNA methylation densities and total contributions from COSMIC signatures in Bie et al. cohort. a)** breast cancer, **b)** colon/rectal cancer, **c)** esophageal cancer, **d)** gastric cancer, **e)** non-small cell lung cancer, and **f)** pancreatic cancer. Each dot represents one sample.


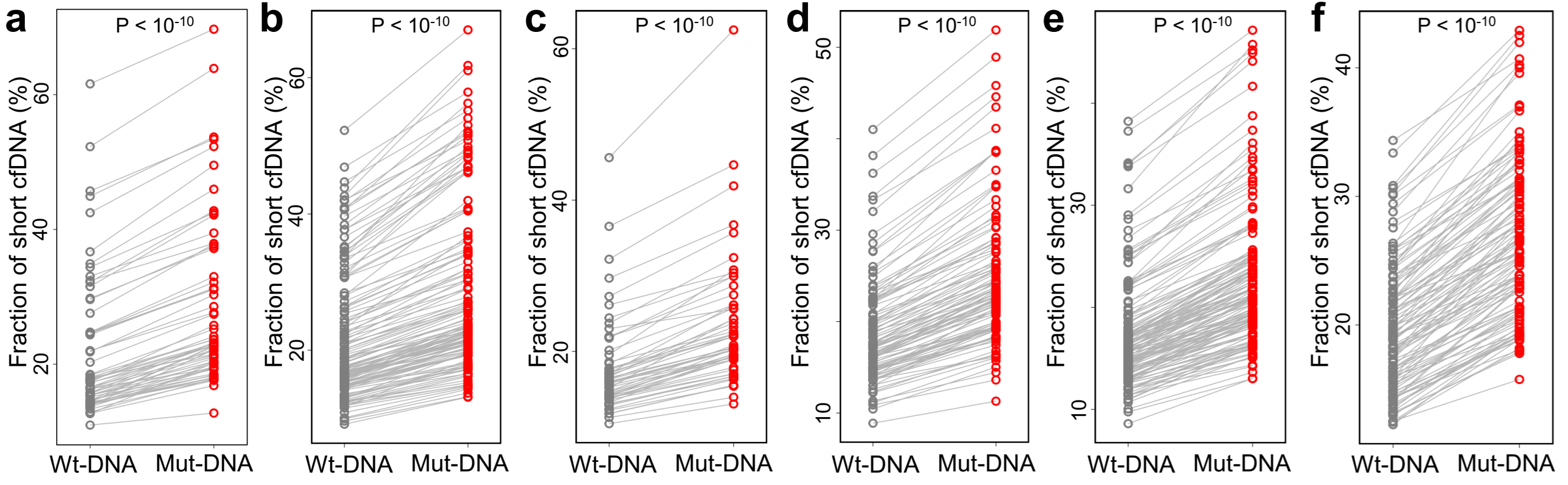


**Fig. S8. Fractions of short cfDNA fragments in Wt- and Mut-DNA in Bie et al. cohort. a)** breast cancer, **b)** colon/rectal cancer, **c)** esophageal cancer, **d)** gastric cancer, **e)** non-small cell lung cancer, and **f)** pancreatic cancer. P-values were calculated using paired t-tests, and each dot represents one sample.


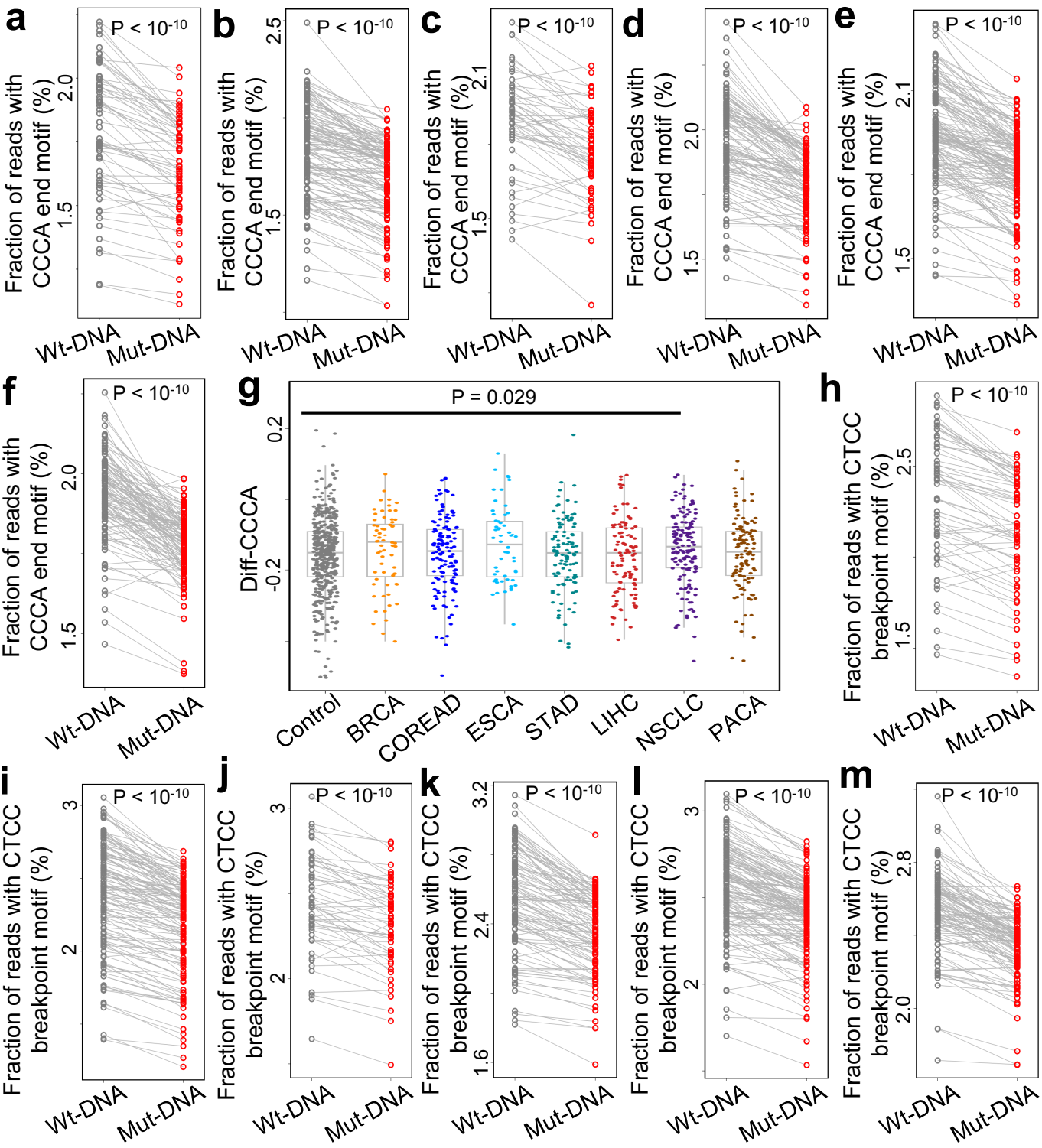


**Fig. S9. End motif usage patterns for Wt- and Mut-DNA in Bie et al. cohort. a-f)** Fraction of reads with CCCA end motif for Wt- and Mut-DNA in **a)** breast cancer, **b)** colon/rectal cancer, **c)** esophageal cancer, **d)** gastric cancer, **e)** non-small cell lung cancer, and **f)** pancreatic cancer. **g)** Diff-CCCA values across controls and cancer samples. **h-m)** Fraction of reads with CTCC breakpoint motif for Wt- and Mut-DNA in **h)** breast cancer, **i)** colon/rectal cancer, **j)** esophageal cancer, **k)** gastric cancer samples, **l)** non-small cell lung cancer, and **m)** pancreatic cancer. In **a-f,h-m**, P-values were calculated using paired t-tests. In **g**, P-values were calculated using Mann-Whitney U test, and Diff-CCCA values showed significant difference in lung cancer samples compared to controls; boxplots represent the median, upper and lower quartiles and whiskers indicate 1.5x IQR. In **a-m**, each dot represents one sample.


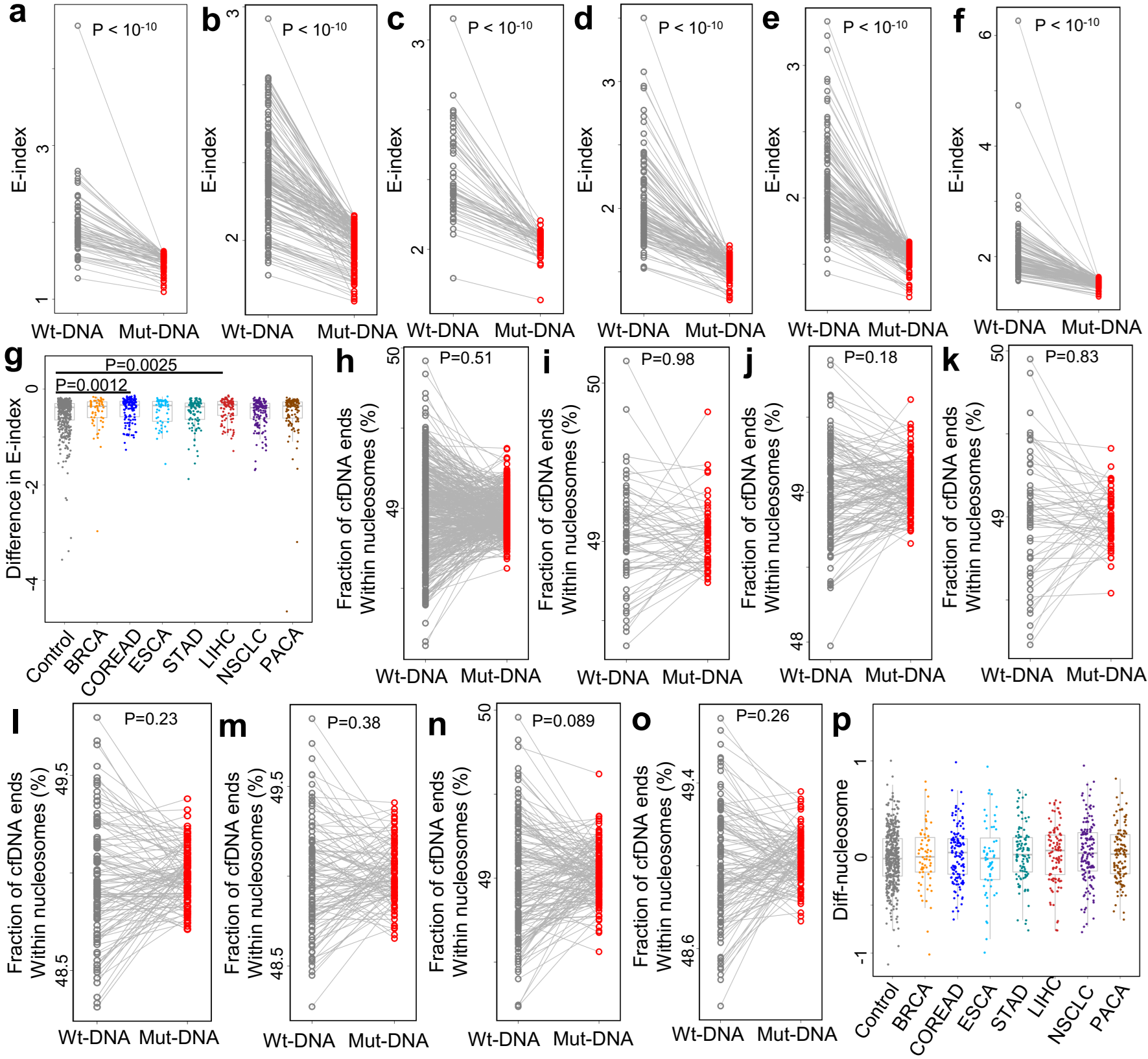


**Fig. S10. Fragment end patterns cfDNA carrying somatic variants in Bie et al. cohort. a-f)** E-index values for Wt- and Mut-DNA in **a)** breast cancer, **b)** colon/rectal cancer, **c)** esophageal cancer, **d)** gastric cancer, **e)** non-small cell lung cancer, and **f)** pancreatic cancer. **g)** Differences in E-index values across controls and cancer samples. **h-o)** Fraction of ends located within nucleosomes for Wt- and Mut-DNA in **h)** controls, **i)** breast cancer, **j)** colon/rectal cancer, **k)** esophageal cancer, **l)** gastric cancer, **m)** liver cancer, **n)** non-small cell lung cancer, and **o)** pancreatic cancer. **p)** Diff-nucleosome values across controls and cancer samples. Each dot represents one sample. In **a-f,h-o**, p-values were calculated using paired t-tests. In **g,p**, p-values were calculated using Mann-Whitney U test. In **p**, Diff-nucleosome values were not significantly different in cancer samples compared to controls. In **g,p**, boxplots represent the median, upper and lower quartiles and whiskers indicate 1.5x IQR.


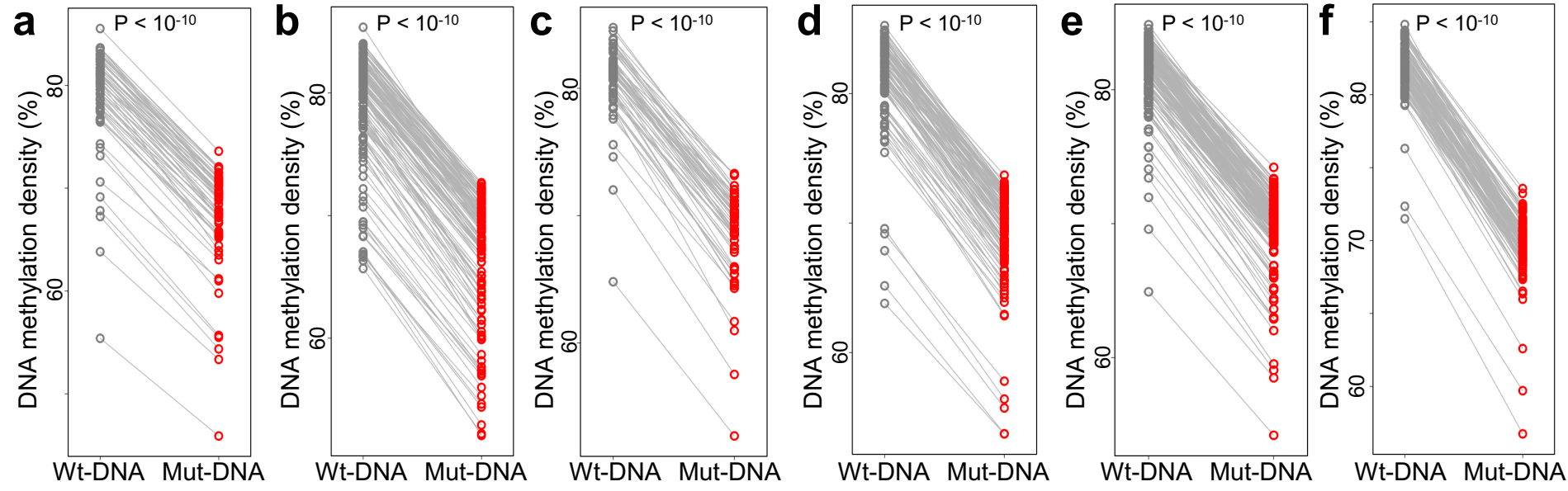


**Fig. S11. DNA Methylation levels for CpGs in gene bodies in Wt- and Mut-DNA in Bie et al. cohort. a)** breast cancer, **b)** colon/rectal cancer, **c)** esophageal cancer, **d)** gastric cancer, **e)** non-small cell lung cancer, and **f)** pancreatic cancer. P-values were calculated using paired t-tests, and each dot represents one sample.


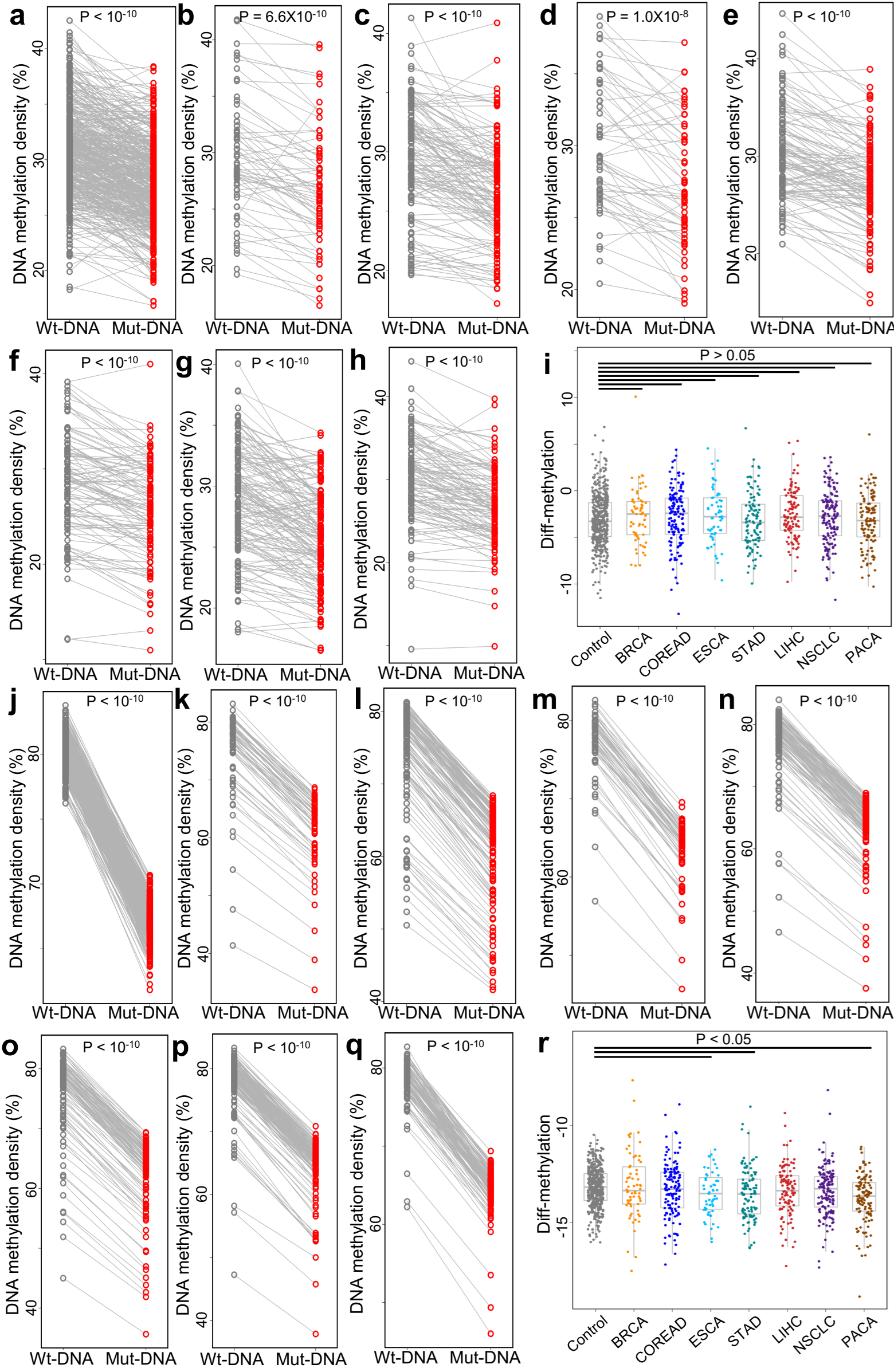


**Fig. S12. DNA Methylation levels for CpGs in promoters and intergenic regions in Wt- and Mut-DNA in Bie et al. cohort. a-i)** For CpGs in promoters, DNA methylation densities in **a)** controls, **b)** breast cancer, **c)** colon/rectal cancer, **d)** esophageal cancer, **e)** gastric cancer, **f)** liver cancer, **g)** non-small cell lung cancer, **h)** pancreatic cancer, and **i)** differences in methylation levels (Diff-methylation) between Mut- and Wt-DNA across controls and cancer samples. **j-r)** For CpGs in intergenic regions, DNA methylation densities in **j)** controls, **k)** breast cancer, **l)** colon/rectal cancer, **m)** esophageal cancer, **n)** gastric cancer, **o)** liver cancer, **p)** non-small cell lung cancer, **q)** pancreatic cancer, and **r)** Diff-methylation between Mut- and Wt-DNA across controls and cancer samples. In **a-r**, each dot represents one sample. In **a-h,j-q**, p-values were calculated using paired t-tests. In **i** and **r**, p-values were calculated using Mann-Whitney U tests between each type of cancer samples and controls; boxplots represent the median, upper and lower quartiles and whiskers indicate 1.5x IQR.


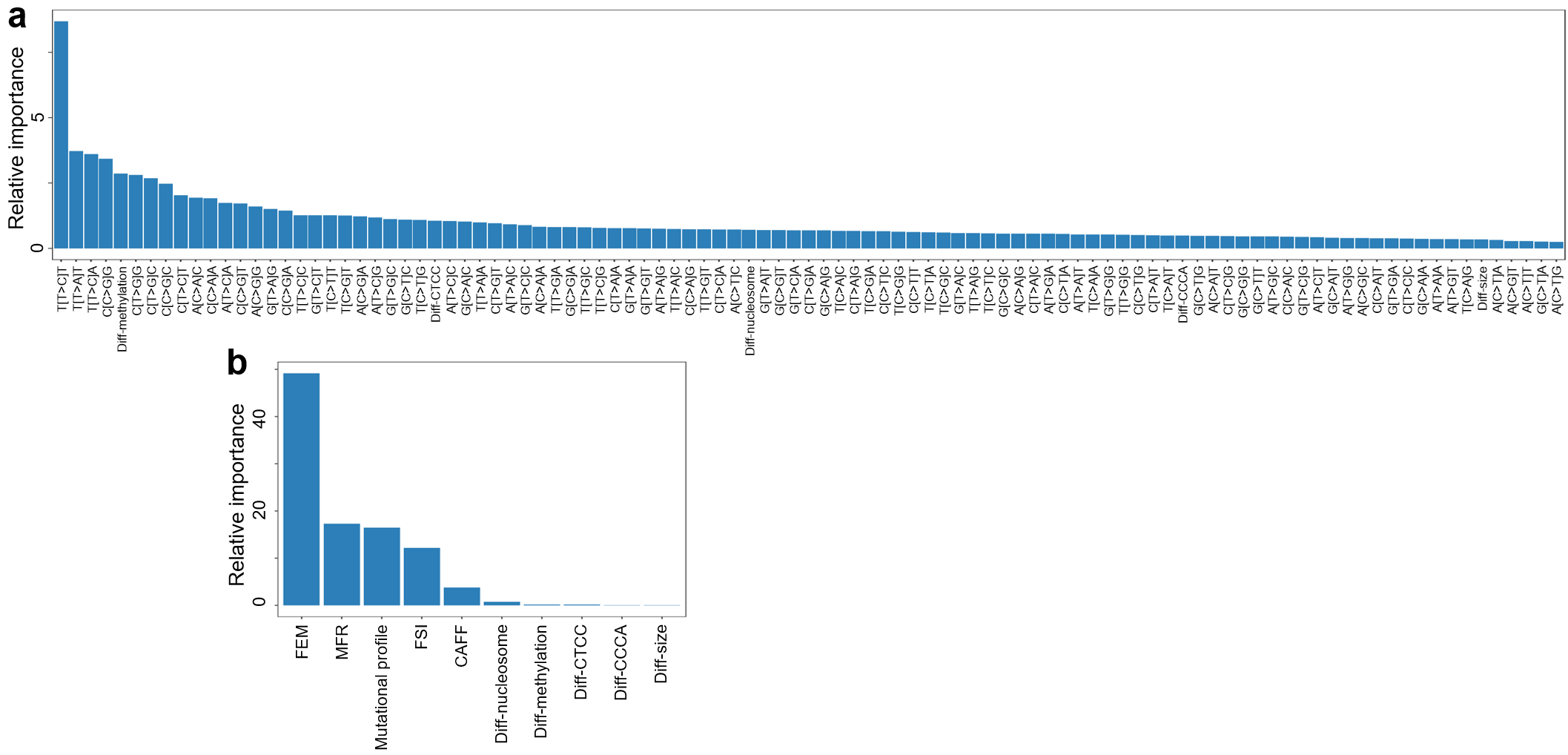


**Fig. S13.** **Relative importance of features in a) FreeSV and b) FreeSV+ models.** The importance scores were extracted during training, and averaged scores for each feature were reported. In b), the features of the same category were grouped.


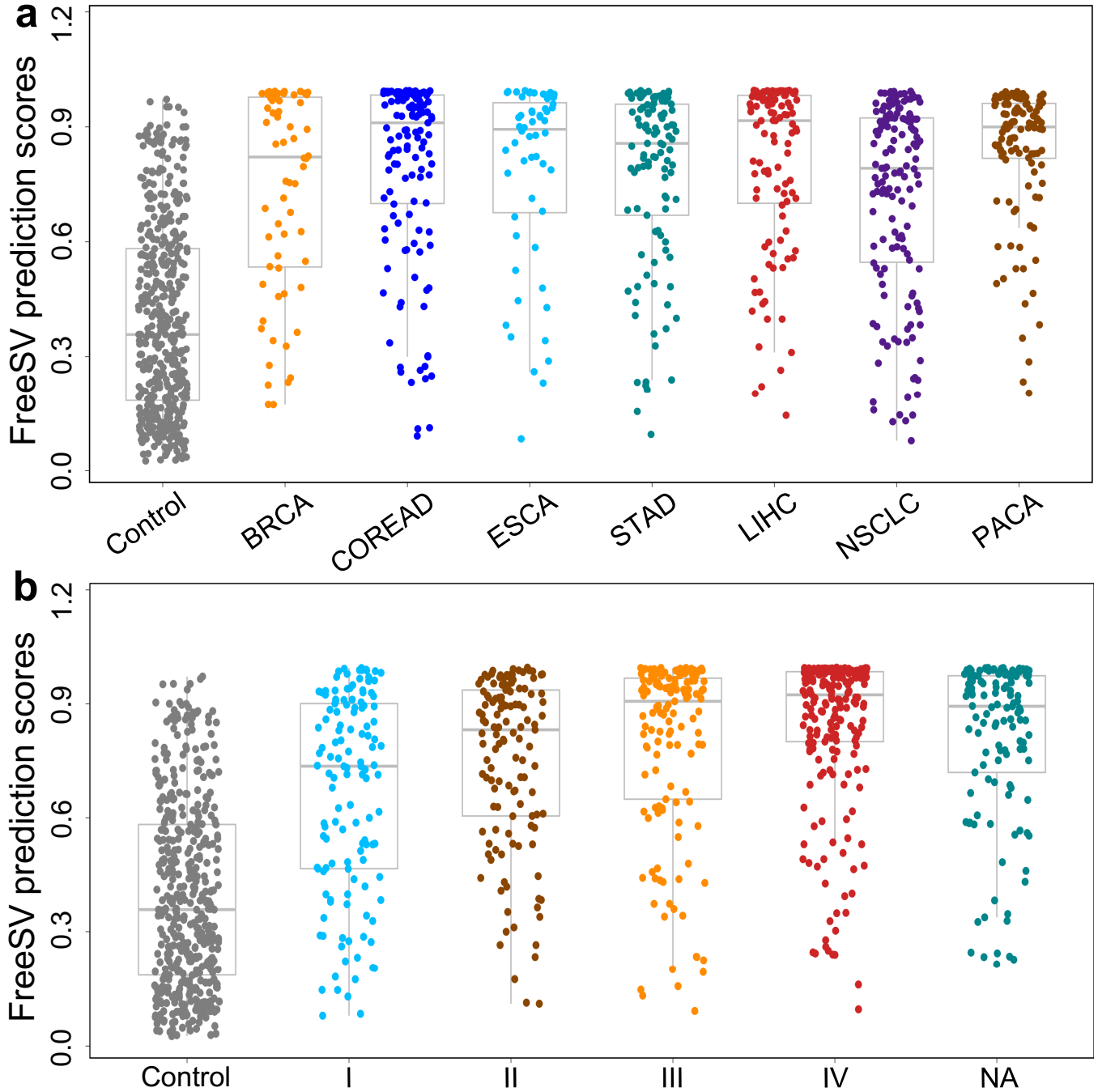


**Fig. S14. Distribution of FreeSV prediction scores across a) cancer types and b) stages.** P-values were calculated using Mann-Whitney U test. Each dot represents one sample and boxplots represent the median, upper and lower quartiles and whiskers indicate 1.5x IQR.


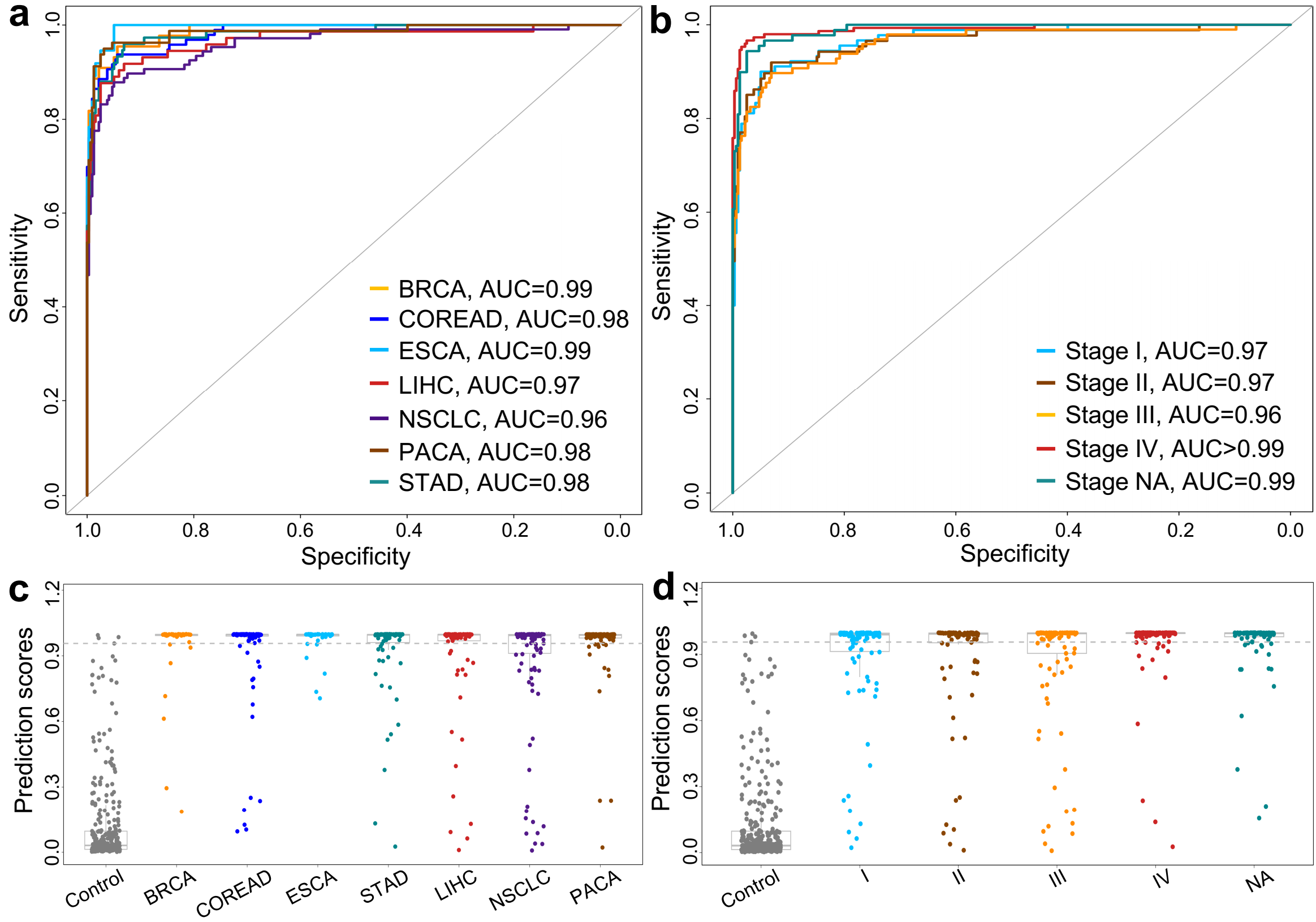


**Fig. S15. Performance of FreeSV+ model on samples in training group in Bie et al. dataset. a-b)** ROC curves across **a)** cancer type**, b)** clinical stage. **c-d)** Distribution of prediction scores across **c)** cancer type, **d)** clinical stage. In **a,b**, P<10^-10^ for all AUCs calculated using Z-tests. In **c,d**, the prediction scores were significantly higher in all cancer samples of different types or stages compared to controls (all P < 10^-10^, Mann-Whitney U tests); the dotted line indicates a threshold with 99% specificity; each dot represents one sample and boxplots represent the median, upper and lower quartiles and whiskers indicate 1.5x IQR.


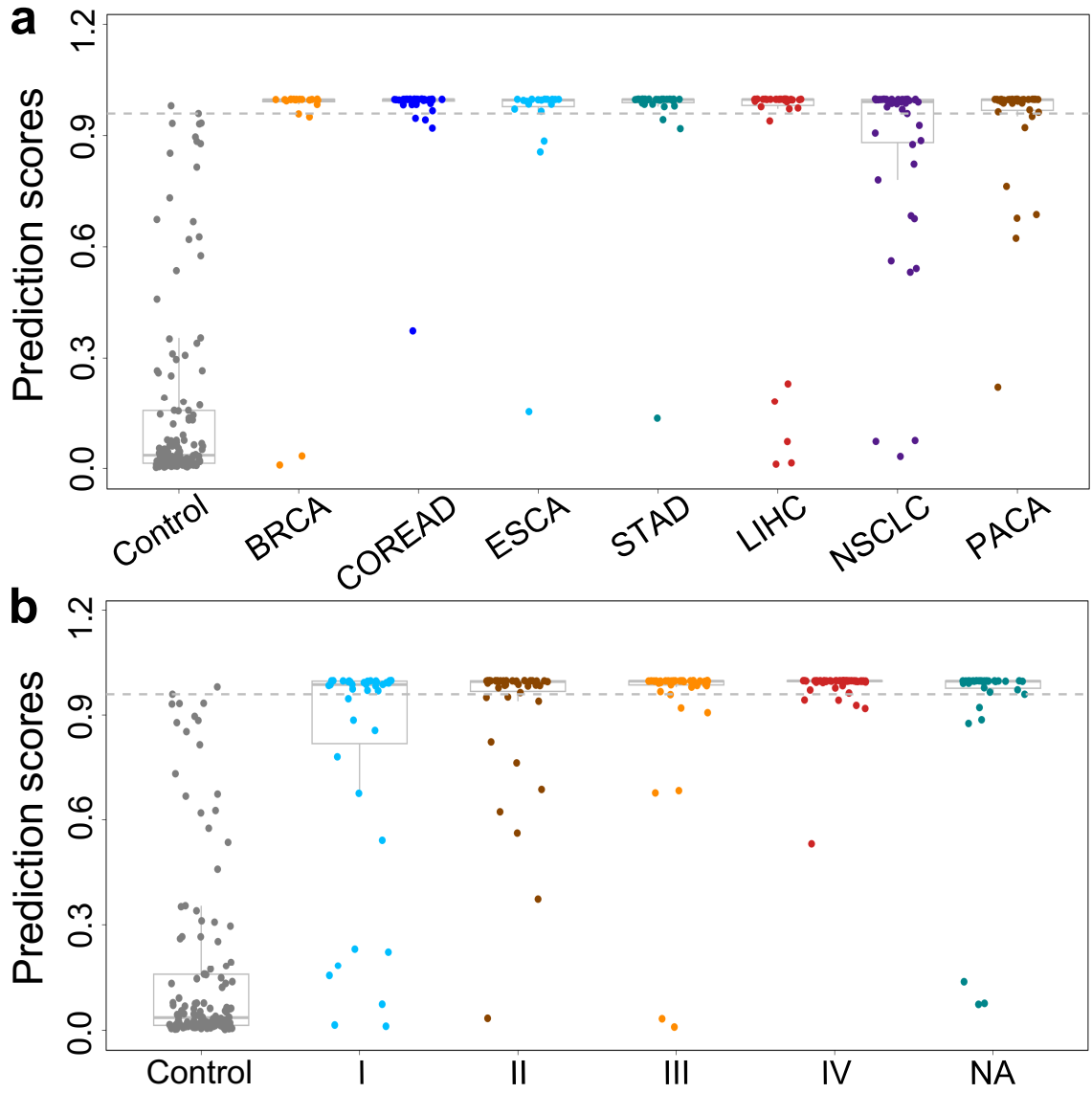


**Fig. S16. Prediction scores of FreeSV+ model on samples in testing group in Bie et al. dataset across a) cancer types, and b) stages.** The prediction scores were significantly higher in all cancer samples of different types or stages compared to controls (all P < 10^-10^, Mann-Whitney U tests). The dotted line indicates a threshold with 99% specificity. Each dot represents one sample and boxplots represent the median, upper and lower quartiles and whiskers indicate 1.5x IQR.
